# Different forms of cardiomyocyte death in post-myocardial infarction ventricular remodeling: mechanisms and therapeutic strategies

**DOI:** 10.3389/fcvm.2026.1797182

**Published:** 2026-06-24

**Authors:** Zhixin Wang, Feng Zhang, Shilong Cao, Xinyu Xue, Hanbing Li, Qian Liu, Dong Wang, Yuting Wu

**Affiliations:** 1 Department of Cardiology, Shandong Medical and Pharmaceutical University Hospital, Binzhou, China; 2 Department of Traditional Chinese Medicine, Shandong Medical and Pharmaceutical University Hospital, Binzhou, China

**Keywords:** cardiomyocyte death, myocardial infarction, non-programmed cell death, programmed cell death, ventricular remodeling

## Abstract

Myocardial infarction (MI) is one of the leading causes of heart failure and cardiovascular mortality worldwide, with post-infarction ventricular remodeling serving as a central pathophysiological mechanism driving the progression toward heart failure, a process that markedly impairs long-term quality of life and contributes to adverse clinical outcomes. Recent studies have gradually elucidated the distinct and overlapping roles of multiple forms of cardiomyocyte death in this process, forming a complex network of programmed cell death; following MI, cardiomyocytes undergo various modes of death including necrosis, apoptosis, as well as more recently identified forms such as ferroptosis and pyroptosis, all of which collectively contribute to ventricular remodeling and the development of heart failure. For instance, ferroptosis, mediated by iron-dependent lipid peroxidation, is markedly elevated after ischemia–reperfusion injury, and inhibition of Glutathione Peroxidase 4 (GPX4) exacerbates plasma membrane rupture, thereby accelerating ventricular wall thinning and left ventricular dilation. Pyroptosis, which relies on activation of the (NOD-like receptor family pyrin domain containing 3-Cysteine-aspartic protease 1-Gasdermin D) NLRP3/caspase-1/GSDMD pathway, leads to the release of inflammatory cytokines, intensifies the local inflammatory microenvironment, and promotes myocardial fibrosis and electrophysiological remodeling. This review systematically summarizes the molecular mechanisms, interplay, and recent advances regarding different forms of cardiomyocyte death in post-MI ventricular remodeling, with particular emphasis on potential therapeutic strategies targeting specific cell death modalities, aiming to provide a novel theoretical foundation and clinical perspectives for optimizing cardioprotection after myocardial infarction.

## 1 Introduction

Acute myocardial infarction (AMI) is often complicated by ventricular remodeling, which further progresses to heart failure (HF) (1, 2). As the most severe cardiovascular disorder, it constitutes a leading cause of mortality worldwide. Ventricular remodeling, the core pathological process underlying heart failure post-AMI, synergistically induces cardiomyocyte death in diverse patterns and degrees via mechanical stress, neurohormonal activation, and inflammatory-immune responses (3). This ultimately results in structural alterations of the left ventricle and impaired cardiac pump function, and this process represents a critical determinant of long-term prognosis in patients.

Previous studies on cell death have been mainly confined to classical apoptosis and necrosis (4). However, with the in-depth advancement of molecular biology and cell death research, a growing body of studies have revealed that cardiomyocyte death subsequent to myocardial infarction involves multiple interconnected forms of programmed cell death (PCD) with distinct morphologies and molecular mechanisms, including apoptosis, necroptosis, pyroptosis, ferroptosis (5), and autophagy-related cell death (6). In recent years, as research progresses continuously, more novel forms of cell death have been uncovered, such as cuproptosis induced by copper ion homeostasis imbalance, and PANoptosis, a complex inflammatory cell death network that integrates the characteristics of apoptosis, necroptosis, and pyroptosis (7, 8). These death forms vary in time, space, and mechanism, collectively forming an intricate cardiomyocyte death network. Studies have indicated that there exist complex interactions between different death forms, which synergistically promote the process of ventricular remodeling following MI. In-depth understanding of the mechanisms underlying these distinct cardiomyocyte death modes and exploration of the crosstalk between them are of great significance for developing targeted therapeutic strategies, interrupting the critical link between AMI and HF, and improving patient prognosis (9). Therefore, this review aims to systematically collate the mechanisms and research progress of different cardiomyocyte death forms in ventricular remodeling after MI, so as to provide new insights for clinical treatment and basic research (10).

## 2 An overview of cardiomyocyte death modalities

### 2.1 Distinct modes of cardiomyocyte demise

Cell death following myocardial infarction primarily encompasses various forms including necrosis, apoptosis, ferroptosis, and pyroptosis. Cell necrosis is triggered by severe pathological stimuli (such as ischemia and calcium overload), leading to ATP depletion and disruption of membrane integrity. The mitochondrial permeability transition pore (mPTP) remains open continuously, resulting in cell swelling, rupture, and the release of damage-associated molecular patterns (DAMPs) (11). Necroptosis is mediated by the RIPK1/RIPK3 - MLKL pathway. After oligomerization, mixed lineage kinase domain-like protein (MLKL) inserts into the membrane to form pores, leading to cell membrane rupture, content leakage, and the release of DAMPs (12). Apoptosis, a programmed cell death process (13), is a form of programmed cell death mediated by the caspase family. It is manifested as the permeabilization of the outer mitochondrial membrane or the activation of death receptors, triggering a cascade reaction that leads to cell shrinkage, nuclear fragmentation, and ultimately the formation of apoptotic bodies that are phagocytosed without triggering inflammation (14). In recent years, novel forms of cell death have been identified, including ferroptosis driven by iron-dependent lipid peroxidation (15), pyroptosis which is caspase-1-dependent and accompanied by substantial release of inflammatory cytokines (16), as well as autophagic cell death (17).

### 2.2 Association with ventricular remodeling

Different forms of cardiomyocyte death exhibit distinct patterns during ventricular remodeling, as summarized in Table 1. Necrotic cells release damage-associated molecular patterns, which initiate inflammatory responses, activate fibroblasts, and promote collagen deposition and fibrosis (18). Apoptosis, mediated via the extrinsic death receptor pathway and the intrinsic mitochondrial pathway with caspases as the central executors, leads to programmed cardiomyocyte death without triggering a robust inflammatory reaction (19). Studies have shown that cardiomyocyte death occurs primarily through necrosis and apoptosis, both playing significant roles in ventricular remodeling (20, 21). Ferroptosis contributes to myocardial injury expansion through the accumulation of lipid peroxidation, resulting in cell membrane damage (22, 23). Pyroptosis, characterized by Gasdermin D (GSDMD) cleavage by caspases to form plasma membrane pores, leads to cell swelling and rupture, releasing inflammatory cytokines such as IL-1β and IL-18, thereby exacerbating local and systemic inflammatory responses and accelerating the progression of ventricular remodeling (16, 24).

**Table 1:** Major forms of cell death and their characteristics following myocardial infarction.

Form of cell death	Primary inducers	Key molecules	Morphological features	Impact on remodeling
Necrosis	Severe Ischemia/ATP Depletion	mPTP	Cellular Swelling/Membrane Rupture	Potent Inflammatory Response/Fibrosis Activation
Necroptosis	Death receptor activation/Ischemia-reperfusion	RIPK1/RIPK3/MLKL	Organelle swelling/Plasma membrane rupture	Cell loss/Exacerbated fibrosis
Apoptosis	Mild Ischemia/Oxidative Stress	Caspase-3/Bax/Bcl-2	Cellular Shrinkage/Chromatin Condensation	Cardiomyocyte Loss/Ventricular Dilation
Ferroptosis	Glutathione Depletion/GPX4 Inhibition	GPX4/ACSL4/LOX	Mitochondrial Shrinkage/Increased Membrane Density	Lipid Peroxidation/Cellular Membrane Damage
Pyroptosis	Inflammasome Activation	Caspase-1/GSDMD	Pyroptotic Cell Swelling/Pore Formation	Intense Inflammatory Response
Autophagy	Excessive Autophagy	LC-3	Formation of Numerous Autophagosomes	Severe Organelle Damage
Cuproptosis	Copper Ion Overload	DLAT/FDX1/LIAS	Loss of Fe-S cluster/Lipoylated DLAT aggregation	Severe Oxidative Stress

## 3 Regulatory mechanisms of distinct cell death modalities and their roles in mediating post-myocardial infarction ventricular remodeling

### 3.1 Necrosis

#### 3.1.1 Overview of the regulatory mechanisms of necrosis

Necrosis is a form of cell death characterized by organelle and plasma membrane disruption, often accompanied by an inflammatory response, and was traditionally regarded as a passive, accidental, and unregulated cellular process (25). The molecular mechanism of necrosis begins with the acute depletion of ATP triggered by severe insults such as ischemia, leading to the functional failure of ion pumps (e.g., Na⁺/K⁺-ATPase and Ca^2^⁺-ATPase) (26), which results in intracellular Na⁺ accumulation, cellular edema, and lethal calcium overload. The energy crisis coupled with calcium overload collectively induces the persistent opening of the mitochondrial permeability transition pore, causing the collapse of the mitochondrial membrane potential, cessation of oxidative phosphorylation accompanied by a burst of reactive oxygen species (ROS), and ultimately leading to mitochondrial swelling, rupture, and the release of its contents. The sustained ion imbalance and osmotic pressure changes result in the complete loss of plasma membrane integrity, culminating in cellular disintegration and the release of large amounts of damage-associated molecular patterns that trigger a severe inflammatory response (27).

#### 3.1.2 Role of necrosis in mediating post-myocardial infarction ventricular remodeling

Myocardial cell necrosis, a critical pathological event in the progression of cardiovascular diseases, directly drives the pathological process of ventricular remodeling by triggering a series of complex molecular and cellular cascades. When cardiomyocytes undergo irreversible necrosis due to factors such as ischemia, oxidative stress, or calcium overload, the loss of cellular membrane integrity leads to the release of intracellular contents, including cardiac troponins and lactate dehydrogenase, into the extracellular microenvironment (28, 29). These damage-associated molecular patterns not only activate the innate immune system, inducing a local inflammatory response (30) and recruiting inflammatory cells, such as neutrophils and macrophages which secrete large quantities of pro-inflammatory cytokines (e.g., TNF-α, IL-1β) and matrix metalloproteinases (MMPs) (31), thereby degrading the extracellular matrix (ECM) and disrupting the structural integrity of the myocardial skeleton, but also directly reduce local contractile units due to the functional loss of necrotic cardiomyocytes. Consequently, the remaining viable cardiomyocytes undergo pathological hypertrophy, disorganized arrangement, and altered electrophysiological properties in response to compensatory functional overload (32). This process is accompanied by the activation and abnormal proliferation of fibroblasts, which transform into myofibroblasts that extensively synthesize and deposit type I and III collagen fibers, leading to interstitial fibrosis. Ultimately, these changes result in abnormal ventricular wall stress distribution, wall thinning or focal thickening, and a geometric transformation of the cardiac chamber from elliptical to spherical, characterized by ventricular dilation and progressive deterioration of systolic function. This maladaptive structural remodeling further exacerbates cardiac pumping dysfunction, establishing a vicious cycle of “necrosis-inflammation-fibrosis-remodeling”, which serves as a crucial pathological foundation for the development and progression of heart failure (33).

### 3.2 Necroptosis

#### 3.2.1 Overview of the regulatory mechanisms of necroptosis

Necroptosis is a tightly regulated, pro-inflammatory mode of cell death whose core mechanism relies on the interaction between receptor-interacting protein kinase 1 and 3 (RIPK1-RIPK3) to form the necrosome, which subsequently phosphorylates and activates mixed lineage kinase domain-like protein (34). Activated MLKL oligomerizes and translocates to the plasma membrane, disrupting membrane integrity and leading to cell lysis (35). This pathway is initiated when apoptosis is inhibited (e.g., in the absence of caspase-8 activity or in the presence of caspase inhibitors) and is finely regulated by the ubiquitination/deubiquitination balance of RIPK1 (modulated by molecules such as CYLD and A20), post-translational modifications of RIPK3 (e.g., phosphorylation and acetylation), and cellular metabolic status (e.g., glutaminolysis) (36). Furthermore, interferon, Toll-like receptor signaling, and certain viruses (e.g., influenza virus) can directly activate the RIPK3-MLKL axis, giving rise to RIPK1-independent alternative pathways. Necroptosis plays a critical role in myocardial ischemia-reperfusion injury, the formation of the necrotic core in atherosclerotic plaques, and inflammatory remodeling in heart failure, making targeting of RIPK3 or MLKL a potential interventional strategy for cardiovascular diseases.

#### 3.2.2 Role of necroptosis in mediating post-myocardial infarction ventricular remodeling

Necroptosis plays a dual-aggravating core role in post-myocardial infarction ventricular remodeling: extensive necroptosis of cardiomyocytes in the infarct zone directly leads to cell loss and scar formation, while simultaneously, the released damage-associated molecular patterns activate the TLR4/NF-*κ*B pathway, thereby inducing a robust sterile inflammatory response that promotes the transition of macrophages toward a profibrotic M2 phenotype and upregulates matrix metalloproteinase activity, resulting in extracellular matrix degradation and an imbalance in interstitial collagen deposition. In the non-infarct zone, persistent oxidative stress and mechanical stress cause aberrant activation of the RIPK1-RIPK3-MLKL pathway, inducing fibroblast proliferation and transdifferentiation into myofibroblasts, which exacerbates reactive fibrosis and increases myocardial stiffness. Of critical importance, necroptosis exhibits cross-regulation with autophagy: impaired autophagy leads to accumulation of damaged mitochondria and reactive oxygen species, further activating RIPK3; conversely, moderate inhibition of necroptosis markedly improves ventricular dilation, reduction in ejection fraction, and inflammatory cell infiltration, suggesting that targeting this pathway holds promise for delaying the progression from post-infarction remodeling to heart failure (37).

### 3.3 Apoptosis

#### 3.3.1 Overview of the regulatory mechanisms of apoptosis

Apoptosis, the most extensively studied form of programmed cell death, originates from 19th-century observations of phenomena such as “chromatolysis” (38) and was formally defined in 1972 by John Kerr and colleagues to distinguish it from necrosis, a process that induces inflammation (39). This process represents a genetically regulated, active, and orderly self-destruction mechanism initiated by specific signals, characterized by the absence of cellular content leakage, thereby typically avoiding the induction of an inflammatory response in surrounding tissues (40).

Morphologically, apoptotic cells exhibit characteristic features including cellular shrinkage, chromatin condensation, and eventual fragmentation into membrane-enclosed “apoptotic bodies”, which are rapidly recognized and phagocytically cleared by macrophages or neighboring cells (41–43).

**Figure 1:**
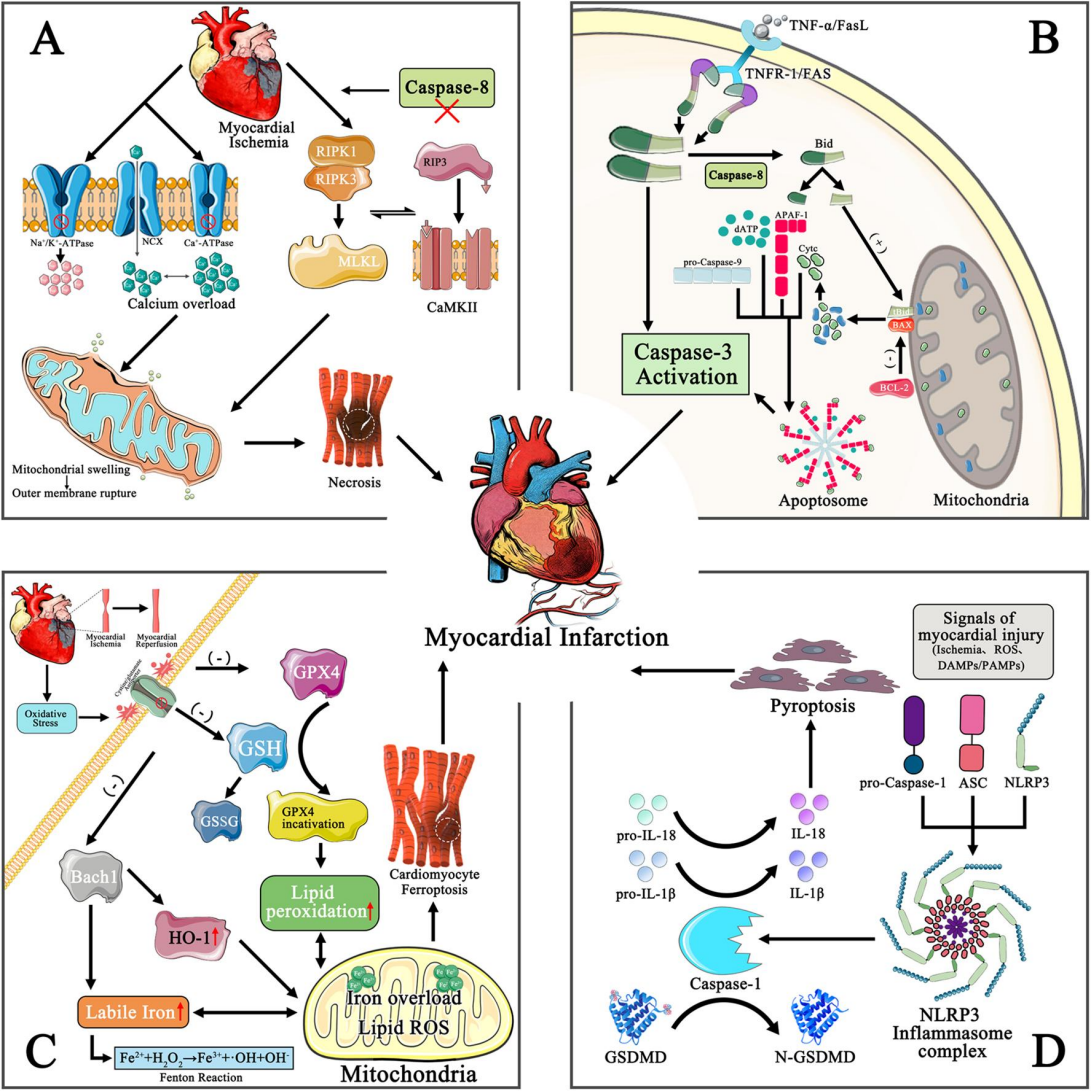
The mechanisms of cell necrosis, apoptosis, ferroptosis, and pyroptosis **(A)** necrosis mechanism: severe myocardial ischemia reduces ATP production, causing intracellular calcium overload that induces mitochondrial swelling and rupture, leading to cardiomyocyte necrosis. **(B)** Apoptosis Mechanism: Mild myocardial ischemia activates Caspase-8, promoting tBid- and BAX-mediated cytochrome c release from mitochondria; Cytc then binds APAF-1, dATP, and pro-Caspase-9 to form the apoptosome, activating Caspase-3/7 and inducing apoptosis. Caspase-8 inhibition shifts the pathway to necroptosis, activating PIPK1-PIPK3-MLKL/CaMKII signaling to directly trigger mitochondrial rupture. **(C)** Ferroptosis Mechanism: In myocardial ischemia/reperfusion injury, ROS generation inhibits system Xc⁻, reducing GPX4 activity and GSH levels while impairing Bach1 function, resulting in lipid peroxidation and labile iron accumulation; iron overload subsequently causes mitochondrial dysfunction and ferroptosis. **(D)** Pyroptosis Mechanism: Danger signals like ischemia activate NF-*κ*B, inducing NLRP3 inflammasome assembly from pro-Caspase-1, ASC, and NLRP3; activated Caspase-1 cleaves GSDMD, pro-IL-18, and pro-IL-1β to generate N-GSDMD pores and mature cytokines, driving inflammatory cytokine release and pyroptosis.

The molecular execution of apoptosis primarily relies on two canonical signaling pathways, namely the extrinsic and intrinsic pathways, as illustrated in Figure 1B (44). The extrinsic pathway, also referred to as the death receptor pathway, is initiated by the binding of extracellular death ligands, such as TNF-α and FasL, to their corresponding death receptors on the cell membrane, such as TNFR-1 and FAS (45). Upon activation, these receptors recruit adaptor proteins to form the death-inducing signaling complex, which subsequently activates initiator caspases, such as caspase-8, and ultimately effector caspases, such as caspase-3/7. In contrast, the intrinsic pathway, also known as the mitochondrial pathway, is typically triggered by intracellular stress signals, including DNA damage and endoplasmic reticulum stress. The core regulatory mechanism of this pathway involves the dynamic balance among Bcl-2 protein family members: under stress conditions, the upregulation of BH3-only proteins antagonizes the anti-apoptotic functions of proteins like Bcl-2, thereby relieving their inhibition on the pro-apoptotic effector proteins Bax and Bak. Activated Bax and Bak oligomerize and form pores in the mitochondrial outer membrane, leading to mitochondrial outer membrane permeabilization and the subsequent release of apoptotic factors, such as cytochrome c (Cytc). Once in the cytoplasm, cytochrome c binds to Apaf-1 to form the apoptosome, which activates caspase-9 and, in turn, the effector caspase-3/7. Both pathways ultimately converge on the activation of effector caspases. These proteases execute apoptosis by cleaving hundreds of cellular substrates, systematically inducing characteristic morphological changes, including cytoskeletal disintegration and DNA fragmentation, facilitating the formation and packaging of apoptotic bodies, and ultimately leading to the complete clearance of apoptotic cells (46, 47).

#### 3.3.2 Role of apoptosis in mediating post-myocardial infarction ventricular remodeling

Apoptosis exerts a sustained and progressive core deleterious role in the process of post-myocardial infarction ventricular remodeling (48). As a form of programmed cell death, although it does not trigger intense inflammatory responses, apoptosis leads to irreversible loss of cardiomyocytes in the infarct border zone and even in remote myocardium via caspase-dependent signaling pathways, directly reducing contractile units and compromising the mechanical integrity of the ventricular wall (49, 50). This cellular loss induces an abnormal increase in wall stress, which subsequently activates the renin-angiotensin-aldosterone system (RAAS) and sympathetic nervous system (SNS) (51, 52), forming a vicious cycle of “apoptosis → increased load → neuroendocrine activation → further apoptosis”.

Meanwhile, various forms of cardiomyocyte death (including apoptosis, necrosis, and necroptosis, among others) can disrupt intercellular junctions and the extracellular matrix network, thereby impairing the uniformity of electrical conduction (53). Although the clearance of apoptotic bodies is relatively “silent” compared to necrosis, it can indirectly drive fibroblast activation and excessive collagen deposition through the release of signaling molecules such as transforming growth factor-*β*, promoting reparative fibrosis and increasing myocardial stiffness (54–56). Ultimately, through multifaceted mechanisms, including direct cardiomyocyte depletion, amplification via mechanical and neuroendocrine feedback loops, disruption of myocardial structural integrity, and the creation of a profibrotic microenvironment, apoptosis collectively drives progressive ventricular dilatation, a geometric shift towards spherical chamber morphology, and both systolic and diastolic dysfunction, thereby steadily and potently promoting the pathological progression toward heart failure in the chronic phase (57).

### 3.4 Ferroptosis

#### 3.4.1 Overview of the regulatory mechanisms of ferroptosis

Ferroptosis, a recently identified form of iron-dependent, non-apoptotic programmed cell death, is characterized by the abnormal accumulation of iron-dependent lipid reactive oxygen species, and is closely associated with dysregulated iron metabolism and the process of lipid peroxidation (58–61). Its core mechanism involves a vicious cycle between the collapse of the antioxidant defense system and a lipid peroxidation storm (62, 63). As specifically illustrated in Figure 1C: upstream stressors (e.g., ischemia/reperfusion, oxidative stress) inhibit the function of the cystine/glutamate antiporter on the cell membrane, leading to reduced uptake of cystine, a critical precursor for glutathione (GSH) synthesis (64), and subsequent glutathione depletion. This results in the inactivation of glutathione peroxidase 4, a key antioxidant enzyme that utilizes glutathione as a cofactor. Once GPX4 is inactivated, its substrates, namely phospholipid hydroperoxides within membrane phospholipids, cannot be adequately reduced and cleared (65).

Concurrently, cardiomyocytes exhibit dysregulated iron metabolism, manifested as increased autophagic degradation of ferritin or impaired iron export, resulting in an expanded intracellular labile iron pool (66, 67). Oxidative stress further promotes the degradation and inactivation of the transcriptional repressor Bach1, thereby relieving its suppression on the heme oxygenase-1 (HO-1) gene, while activating transcription factors such as Nrf2, collectively driving a significant upregulation of HO-1 expression (68). Elevated HO-1 levels accelerate heme degradation, releasing protective byproducts like biliverdin and carbon monoxide but also generating substantial amounts of free ferrous ions, which further expand the labile iron pool (69–72). The abundant free ferrous ions within this pool catalyze the Fenton reaction, converting hydrogen peroxide into highly reactive hydroxyl radicals (73–76), and directly drive the peroxidation cascade of polyunsaturated fatty acids in membrane phospholipids. In the absence of GPX4 activity, the resulting phospholipid hydroperoxides accumulate explosively, compromising the integrity of mitochondrial and plasma membranes (77), and inducing characteristic mitochondrial alterations (e.g., reduced cristae, increased membrane density, and condensation). This cascade ultimately leads to membrane system disintegration and cell death (78, 79).

#### 3.4.2 Role of ferroptosis in mediating post-myocardial infarction ventricular remodeling

Ferroptosis plays a dual pathogenic role as an “amplifier of oxidative damage” and an “engine of chronic inflammation” during post-myocardial infarction ventricular remodeling (80). It not only directly causes irreversible loss of contractile myocardial units, but also establishes a persistent and potent oxidative-inflammatory microenvironment through the release of unique damage signals including oxidized phospholipids, labile iron, and lipid peroxidation metabolites (81). This microenvironment continuously impairs the function of adjacent surviving cardiomyocytes and potently activates the transformation of cardiac fibroblasts into myofibroblasts, thereby driving excessive collagen deposition and interstitial fibrosis while disrupting myocardial electrical conduction stability (82, 83). Consequently, via the tripartite pathological mechanisms of direct cytotoxicity (84), oxidative-inflammatory cascade amplification, and pro-fibrotic action, ferroptosis synergistically promotes increased ventricular wall stiffness, progressive dilation, and systolic dysfunction, comprehensively accelerating adverse ventricular remodeling and heart failure progression in both acute and chronic stages.

### 3.5 Pyroptosis

#### 3.5.1 Overview of the regulatory mechanisms of pyroptosis

Pyroptosis in cardiomyocytes is a programmed form of cell death accompanied by intense inflammatory responses (85), which distinguishes it from apoptosis and necrosis and constitutes a crucial pathological link connecting acute myocardial injury with the progression of chronic heart failure (86). It has emerged as a significant therapeutic target in cardiovascular disease research (87, 88). As illustrated in Figure 1D, the mechanism of cardiomyocyte pyroptosis is initiated by the sensing of danger signals: under pathological conditions such as myocardial ischemia or infection, pattern recognition receptors detect damage-associated molecular patterns (e.g., DAMPs) or pathogen-associated molecular patterns (PAMPs) (89), triggering Signal 1 that activates the NF-*κ*B pathway and subsequently upregulates the expression of key proteins including NLRP3, pro-IL-1*β*, and pro-IL-18 (90–93). Subsequently, secondary signals (Signal 2), mediated by ATP-induced K⁺ efflux, mitochondrial reactive oxygen species burst, or lysosomal rupture, induce conformational changes and oligomerization of NLRP3, which then recruits the adaptor protein ASC via PYD-PYD domain interactions. ASC undergoes prion-like polymerization to form microscopically visible “ASC specks” that recruit large amounts of pro-caspase-1 through CARD-CARD domain interactions, leading to its aggregation, autoproteolysis, and conversion into active caspase-1 (94–96).

Activated caspase-1 cleaves gasdermin D, releasing its N-terminal fragment (97–99), which subsequently oligomerizes on the plasma membrane to form pores that disrupt ionic homeostasis, leading to osmotic swelling, membrane rupture, and pyroptotic cell death (100). Concurrently, caspase-1 processes pro-IL-1*β* and pro-IL-18 into mature inflammatory cytokines (101). The pyroptotic cardiomyocytes release substantial amounts of damage-associated molecular patterns and mature cytokines, triggering a severe inflammatory storm that further damages adjacent viable myocardium, activates cardiac fibroblasts to promote fibrosis, and disturbs electrical conduction, collectively driving the loss of contractile function, adverse ventricular remodeling, and progression toward heart failure.

#### 3.5.2 Role of pyroptosis in mediating post-myocardial infarction ventricular remodeling

In the process of mediating post-myocardial infarction ventricular remodeling, pyroptosis can be characterized as both an “inflammatory storm initiator” and an “immune injury amplifier”. Pyroptosis not only directly leads to cardiomyocyte rupture and death due to osmotic swelling but, more critically, actively triggers an intense local inflammatory storm (102–105). The potent cytokines released thereby recruit and activate a large number of neutrophils and monocytes in large quantities. While clearing necrotic debris, these immune cells release proteases and reactive oxygen species, indiscriminately attacking surrounding viable cardiomyocytes and the extracellular matrix, resulting in “bystander injury” that significantly expands the infarct zone (106, 107). Furthermore, the persistently high-concentration inflammatory microenvironment strongly drives cardiac fibroblast activation, leading to pathological collagen over-deposition and the formation of rigid and electrically heterogeneous fibrotic scars (108).

Through the triple pathological mechanisms of “direct cytotoxicity, inflammatory cascade amplification, and pro-fibrotic repair”, pyroptosis synergistically contributes to ventricular wall thinning, progressive dilation, impairment of both systolic and diastolic functions, and increased susceptibility to arrhythmias, thereby comprehensively accelerating the progression of adverse ventricular remodeling and heart failure (109).

### 3.6 Autophagy

**Figure 2:**
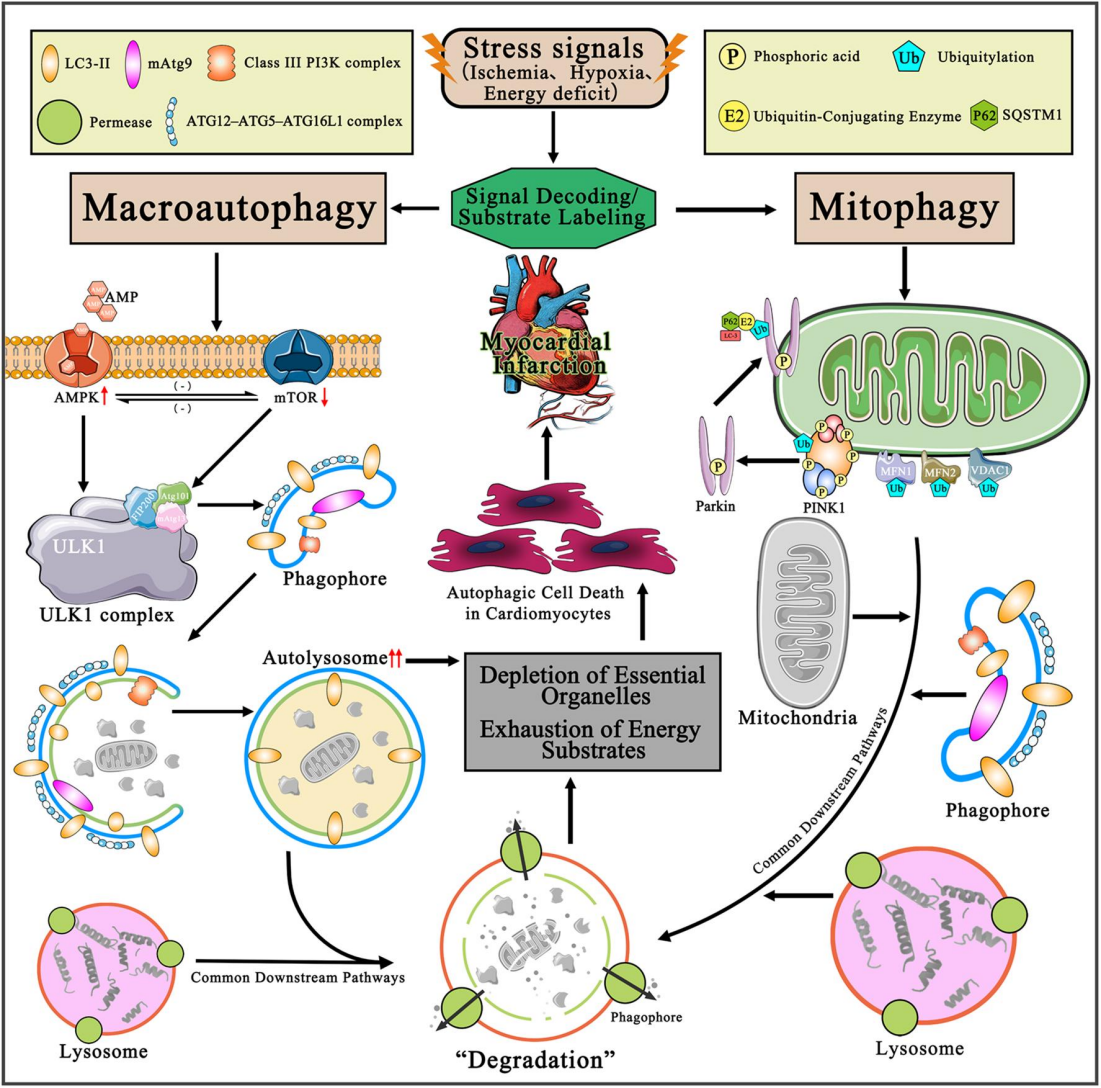
Mechanisms of cellular macroautophagy and mitochondrial autophagy. Cells decode stress signals (such as ischemia or energy deficiency) and tag substrates to initiate either macroautophagy or mitophagy. As illustrated on the left side of the figure, macroautophagy is activated via AMPK stimulation and mTOR inhibition, leading to ULK complex activation an phagophore formation. This engulfs damaged mitochondria and organelles to form an autophagosome, which subsequently fuses with a lysosome to become an autolysosome, degrading its contents. As illustrated on the right side of the figure, mitophagy is mediated through the canonical PINK1/Parkin pathway, in which activated Parkin ubiquitinates damaged mitochondria to generate signals recognized by phagophores. The labeled mitochondria are then engulfed to form autophagosomes, which subsequently fuse with lysosomes to become autolysosomes for degradation. Excessive activation of either macroautophagy or mitophagy can lead to depletion of essential organelles and energy substrates, ultimately resulting in cell death.

Autophagy is a highly conserved self-renewal process in which cells form double-membrane autophagosomes under stress conditions to sequester damaged organelles, protein aggregates, or invading pathogens, and subsequently deliver them to lysosomes for degradation and recycling (110). Its core functions lie in maintaining intracellular homeostasis, providing energy substrates, and clearing harmful components (111, 112). Depending on substrate specificity and molecular mechanisms, autophagy can be primarily classified into non-selective macroautophagy (113) and selective mitochondrial autophagy (mitophagy), which targets damaged mitochondria (114) (as illustrated in Figure 2). This process is tightly regulated by sophisticated signaling networks, such as the AMPK/mTOR/ULK1 axis (115, 116), and plays a dual role in cardiomyocytes: moderate autophagy acts as a protective adaptive mechanism in response to stresses such as ischemia, whereas excessive or insufficient autophagy leads to either excessive depletion of cellular components or accumulation of toxic substances, thereby promoting cardiomyocyte death and exacerbating disease progression, including heart failure (117).

#### 3.6.1 Macroautophagy

The mechanism of macroautophagy initiates when cells perceive stress signals such as energy or nutrient deprivation (118–120), leading to AMPK activation and mTOR inhibition, which subsequently relieves the repression on the ULK1 complex (121, 122). The activated ULK1 complex then phosphorylates and activates the class III PI3 K complex located on membrane structures like the endoplasmic reticulum, catalyzing the generation of phosphatidylinositol 3-phosphate as a critical lipid signal; this recruits effector proteins containing FYVE/PX domains (e.g., WIPI proteins) along with the ATG9A vesicular system to provide membrane sources (123–125). Subsequently, two ubiquitin-like conjugation systems, the ATG12-ATG5-ATG16L1 complex and the LC3 lipidation system, are sequentially activated. Among these, the ATG16L1 complex functions akin to an E3 ligase, facilitating the conjugation of cytosolic LC3-I with phosphatidylethanolamine to form membrane-bound LC3-II, thereby driving the elongation, curvature, and eventual closure of the isolation membrane to form a double-membraned autophagosome (126–128). The mature autophagosome is transported via microtubules to fuse with lysosomes, forming an autolysosome wherein the engulfed cargo is thoroughly degraded by lysosomal hydrolases; the resulting small molecules such as amino acids and fatty acids are released back into the cytoplasm for recycling, thereby accomplishing the complete dynamic process from stress sensing, membrane nucleation, substrate engulfment to degradation and recovery (129–132).

#### 3.6.2 Mitophagy

The mechanism of mitophagy is initiated by mitochondrial damage, with the loss of membrane potential serving as the key initiating signal (133). This change leads to the stabilization and accumulation of the PINK1 protein on the outer mitochondrial membrane, which subsequently phosphorylates ubiquitin molecules and recruits and activates the cytosolic E3 ubiquitin ligase Parkin (134–136). Activated Parkin extensively polyubiquitinates outer mitochondrial membrane proteins (e.g., MFN1/2, VDAC1), generating prominent “eat-me” signals. These ubiquitin chains are then specifically recognized by autophagy adaptor proteins (e.g., OPTN, NDP52) (137, 138). The adaptor proteins, via their LC3-interacting region, simultaneously bind to LC3 proteins on the phagophore membrane, thereby anchoring the damaged mitochondrion to the forming autophagic membrane. This process further recruits and locally activates core autophagy-related proteins (including the ULK1 complex and the phosphatidylinositol 3-phosphate generation system), guiding the specific encapsulation of the tagged mitochondrion by the expanding phagophore, ultimately leading to the formation of a mitophagosome. The mitophagosome subsequently fuses with a lysosome, resulting in the complete degradation of its contents, thereby accomplishing the selective clearance of dysfunctional mitochondria and maintaining cellular metabolic homeostasis and viability (139).

#### 3.6.3 Role of autophagy in mediating post-myocardial infarction ventricular remodeling

The effect of autophagy on ventricular remodeling exhibits a distinct “dual-phase” nature, which is highly dependent on the integrity of the autophagic flux, its activation level, and the specific spatial-temporal context (140). Under conditions of acute ischemia and moderate stress, protective autophagy effectively maintains cardiomyocyte homeostasis, suppresses other cell death pathways such as apoptosis, pyroptosis, and ferroptosis, thereby limiting initial injury and promoting adaptive repair by clearing toxic components including damaged mitochondria and misfolded proteins, while also providing energy substrates like amino acids and fatty acids (141, 142). However, when autophagy is abnormally, persistently, and excessively activated or its downstream degradation steps are impaired, it can shift toward pathological effects: excessively activated autophagy may lead to the excessive degradation of essential organelles and contractile proteins, triggering autophagic cell death (143, 144); whereas a blockage in autophagic flux results in the accumulation of toxic protein aggregates and dysfunctional organelles (145), continuously generating reactive oxygen species and activating secondary death pathways such as apoptosis (146).

In the chronic phase of ventricular remodeling, this dual nature of autophagic function is particularly critical; a moderate and unimpeded autophagic flux is essential for sustaining long-term cardiomyocyte health (147), whereas dysfunctional autophagy profoundly influences the trajectory of ventricular remodeling and the progression of heart failure by directly contributing to cell loss, exacerbating oxidative stress and the inflammatory microenvironment, and indirectly promoting cardiac fibroblast activation along with pathological collagen deposition (140), collectively leading to ventricular wall thinning, fibrotic scar formation, progressive dilation, and deterioration of contractile function (148).

### 3.7 Cuproptosis

#### 3.7.1 Overview of the regulatory mechanisms of cuproptosis

As a newly discovered form of cell death, cuproptosis was proposed by Peter Tsvetkov and colleagues in 2022, who further elucidated its underlying mechanisms (149). Cuproptosis depends on the intracellular accumulation of copper and exhibits distinct morphological features, although the specific cellular morphological changes associated with this process remain unclear and require further investigation (150).

**Figure 3:**
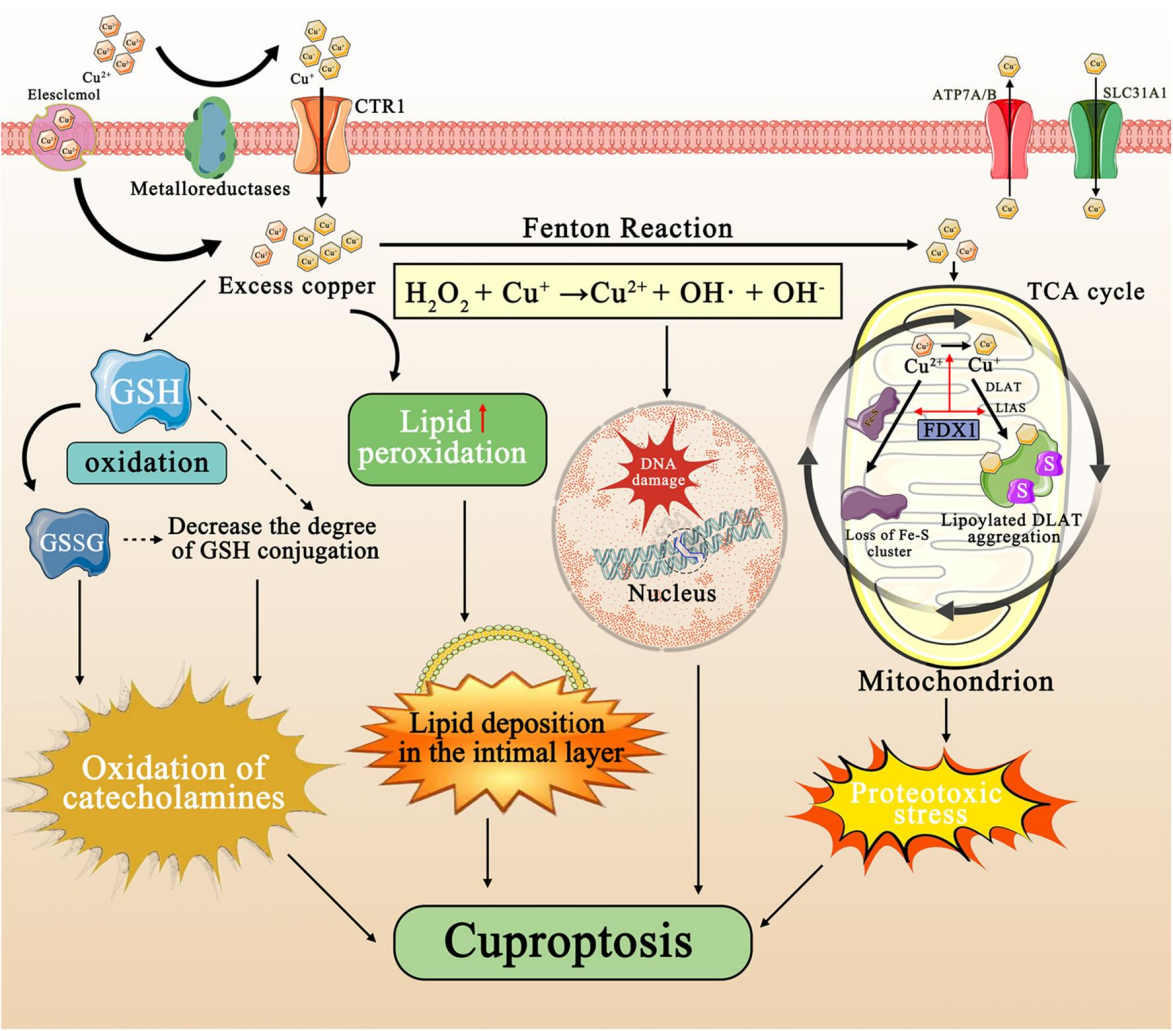
Cuproptosis mechanism. Copper ions enter cells through various pathways, leading to abnormal accumulation and copper overload. This is accompanied by a reduction in antioxidants such as glutathione (GSH) and the generation of free radicals via the Fenton reaction, which induce lipid peroxidation and DNA damage. Furthermore, excess copper directly binds to lipoylated proteins involved in mitochondrial respiration, particularly dihydrolipoamide S-acetyltransferase, resulting in aberrant aggregation of lipoylated proteins and loss of iron-sulfur cluster proteins. These events trigger proteotoxic stress and disrupt mitochondrial respiratory function, ultimately culminating in cell death.

As an essential trace element in the human body, copper participates extensively in various signaling pathways and tumor-related pathophysiological processes (151–153). Unlike other forms of programmed cell death, cuproptosis is primarily driven by the intracellular accumulation of excess copper, rather than by the presence of specific ionophores. As illustrated in Figure 3, elesclomol facilitates the transmembrane transport of divalent copper ions into mitochondria (154, 155), where they directly target mitochondrial ferredoxin 1 and are reduced to the more toxic monovalent copper ions (156). Within mitochondria, the reduced copper ions bind to the TCA cycle key enzyme, dihydrolipoamide S-acetyltransferase (DLAT), which has undergone post-translational lipoylation, disrupting its native conformational stability and leading to abnormal cross-linking and irreversible aggregation of the enzyme (157, 158). Furthermore, copper-induced protein aggregation downregulates the expression and impairs the stability of iron-sulfur cluster proteins, thereby triggering proteotoxic stress and ultimately leading to cell death (155, 159–161). Although the understanding of cuproptosis as a newly identified form of cell death remains relatively limited, studies suggest that targeting this pathway may represent a potential therapeutic strategy for tumors.The aberrant copper metabolism in tumor cells renders them highly susceptible to cuproptosis, which can be induced through strategies such as copper ionophores, nano-drug delivery, and combination with radiotherapy, chemotherapy, or metabolic inhibitors. This provides a novel direction for overcoming drug resistance and achieving precise anticancer effects, positioning cuproptosis as a highly promising therapeutic target in oncology (162). Future investigations are required to further elucidate the regulatory mechanisms and morphological characteristics of cuproptosis, as well as to identify specific inducers and inhibitors of this pathway.

#### 3.7.2 Role of cuproptosis in mediating post-myocardial infarction ventricular remodeling

Intense oxidative stress induced by the ischemia/reperfusion process following myocardial infarction serves as a core pathological driver of ventricular remodeling, wherein dysregulated copper metabolism plays a pivotal role (163). Copper ions can catalytically generate highly toxic hydroxyl radicals via the Fenton reaction cycle, leading to DNA damage, lipid peroxidation, depletion of the glutathione antioxidant system, and the oxidation of catecholamines into products with direct cardiotoxicity (164, 165). Recent studies have revealed that abnormal copper accumulation can trigger a unique form of regulated cell death termed “cuproptosis”, wherein excess copper ions directly attack and induce abnormal aggregation of key lipoylated proteins within the tricarboxylic acid cycle in mitochondria (166), resulting in mitochondrial respiratory chain dysfunction and proteotoxic stress. The upregulation of copper-dependent lysyl oxidase isoforms exacerbates extracellular matrix remodeling and fibrosis (167); although endogenous copper/zinc superoxide dismutase may partially alleviate oxidative damage, the overall disruption of copper homeostasis ultimately contributes, through synergistic mechanisms involving oxidative injury, metabolic failure, and proteotoxicity, to extensive cardiomyocyte loss, fibrotic scar formation, and the decline of cardiac contractile function, thereby significantly accelerating the progression of adverse ventricular remodeling and heart failure (168).

## 4 Crosstalk among distinct cell death pathways

**Figure 4:**
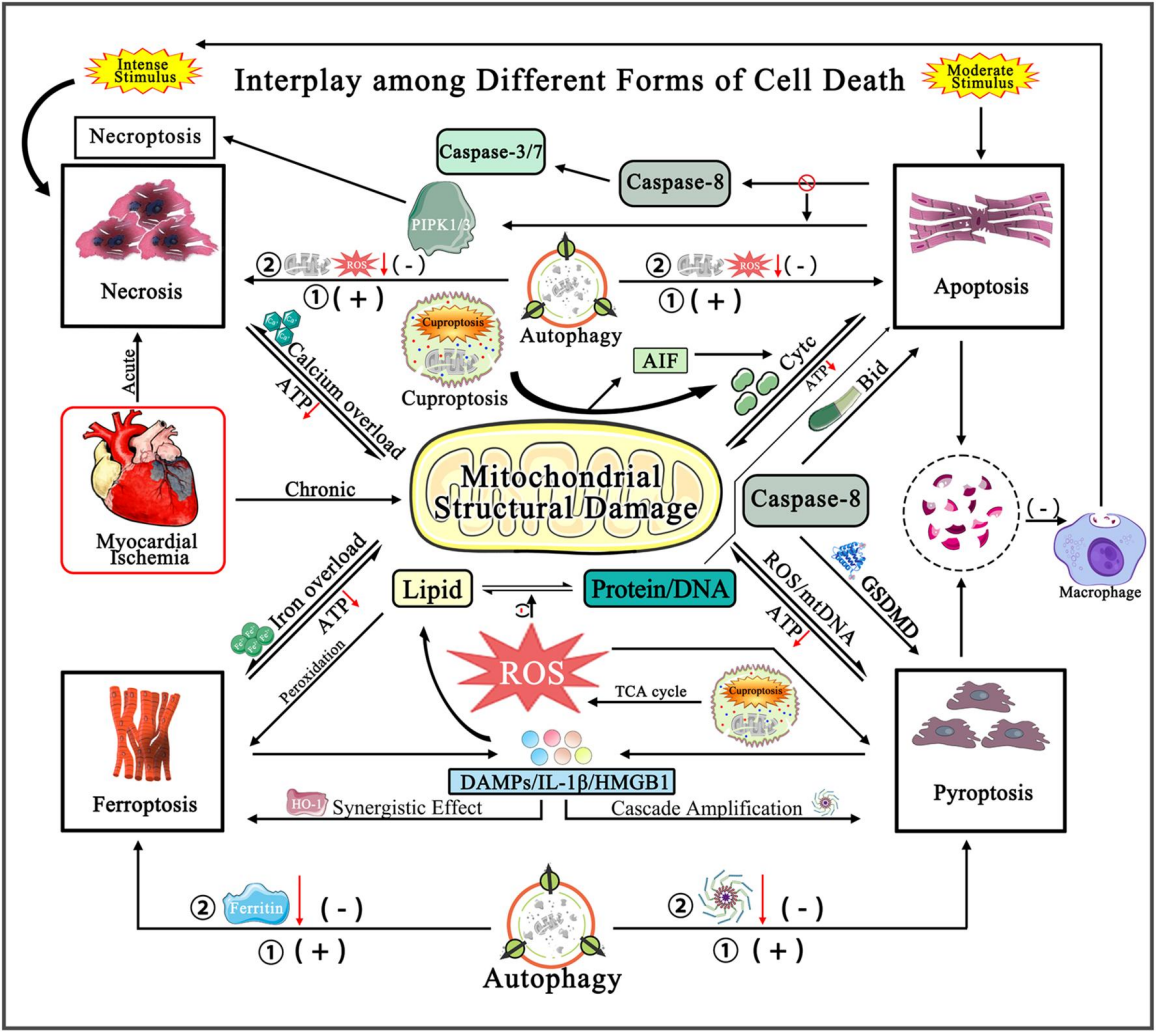
Interplay among different forms of cell death. There exists a closely intertwined bidirectional regulatory relationship between cell death and mitochondrial dysfunction. The occurrence of necrosis, apoptosis, ferroptosis, and pyroptosis can all impair mitochondrial function, leading to reduced ATP production; conversely, mitochondrial damage can further create a positive feedback loop, exacerbating the cell death process. When exogenous factors, such as pharmacological inhibition, suppress Caspase-8 activity, the apoptotic pathway becomes obstructed, and the mode of cell death may shift toward necroptosis. Mild injury typically initiates the apoptotic program; however, if apoptotic cellular debris is not promptly cleared by macrophages, it can trigger an inflammatory storm, converting into a strong stimulus that further activates the necrotic pathway. Additionally, abnormally elevated ROS induced by stimuli can disrupt protein structures and their normal interactions with DNA and lipids, resulting in lipid peroxidation; iron overload, on the other hand, can directly trigger ferroptosis or accelerate pyroptosis.Autophagy, as a crucial process in maintaining intracellular homeostasis, can eliminate damaged organelles and inflammatory factors, as indicated in pathway ②. However, excessive activation of autophagy may instead accelerate inflammatory responses and aggravate cell death, as shown in pathway ①. Cuproptosis, a recently discovered form of cell death, promotes ROS generation through involvement in the tricarboxylic acid cycle, thereby facilitating the occurrence of ferroptosis and pyroptosis; simultaneously, cuproptosis can also damage mitochondria, promote the release of cytochrome c (Cytc) and apoptosis-inducing factor (AIF), and thereby initiate the apoptotic program.

As shown in Figure 4, there exists a tightly intertwined bidirectional regulatory relationship between different forms of cell death and mitochondrial dysfunction (169). Meanwhile, cell death also exhibits time dependence, as presented in Table 2. The occurrence of necrosis, apoptosis, ferroptosis, and pyroptosis can all impair mitochondrial function, leading to reduced ATP production. Conversely, mitochondrial damage further establishes a positive feedback loop that exacerbates the cell death process. During myocardial ischemia–reperfusion injury and subsequent ventricular remodeling, the above-mentioned forms of cell death do not occur randomly but exhibit a clear time-dependent evolution, with different forms predominating at distinct pathological stages and exerting differential effects on arrhythmogenic substrate formation and cardiac remodeling (170).

**Table 2:** Distinct cardiomyocyte death modalities and crosstalk across different phases.

Stage	Core pathology	Major cell death modalities	Key interaction effect
Ischemia Period	Hypoxia,energy depletion,calcium overload	Necrosis, necroptosis; protective autophagy	Autophagy inhibits necrosis; apoptosis, ferroptosis, pyroptosis and cuproptosis are suppressed
Early Reperfusion	ROS burst, mitochondrial damage	Apoptosis, ferroptosis predominance; pyroptosis and cuproptosis initiated	ROS cross-activates multiple death pathways; autophagy presents bidirectional regulation
Late Reperfusion	Inflammatory storm, DAMPs release	Pyroptosis, necroptosis dominant; persistent apoptosis and ferroptosis	Inflammatory cascade amplifies cell death; protective autophagy is impaired
Remodeling Period	Inflammation subsided, myocardial fibrosis	Chronic low-level apoptosis and ferroptosis	Acute cell death pathways subsided; protective autophagy alleviates cardiac remodeling

First, during the ischemic phase (characterized by hypoxia/nutrient deprivation), the primary modes of cell death include passive necrosis (predominantly early-stage), initiation of apoptosis, and activation of autophagy. In this phase, severe ATP depletion leads to ion pump failure, cellular edema, acidosis, opening of the mitochondrial permeability transition pore, and calcium overload. Mild injury typically initiates the apoptotic program, but its execution is hindered by insufficient ATP. Autophagy is activated to remove damaged organelles in an attempt to maintain cell survival. When exogenous factors (e.g., pharmacological inhibition) suppress caspase-8 activity, the apoptotic pathway is blocked, and the mode of cell death may shift toward necroptosis (171). The consequences of these events include cell swelling, partial necrotic rupture, release of damage-associated molecular patterns, and initiation of early inflammatory signals, resulting in the loss of cardiomyocytes without yet generating a strong inflammatory storm.

Second, during the early reperfusion phase, the dominant pathways are the initiation of necroptosis, along with persistent apoptosis and necrosis. At this stage, cardiomyocytes exhibit a burst of reactive oxygen species and a sharp exacerbation of calcium overload. Caspase-8 activity is suppressed upon reperfusion, blocking the apoptotic pathway and shifting cell death toward necroptosis. Activation of the RIPK1–RIPK3–MLKL pathway leads to plasma membrane rupture. If apoptotic cell debris is not promptly cleared by macrophages, it can trigger an inflammatory storm, which in turn strongly activates the necrotic pathway. The massive release of DAMPs during this phase induces acute inflammation and activates inflammatory cytokines such as IL-1β, leading to a robust inflammatory response (172). Disruption of gap junctions and impairment of electrical conduction homogeneity establish an arrhythmogenic substrate, while cardiomyocyte death dramatically escalates, marking the core period of reperfusion injury.

Third, the late reperfusion phase is characterized by pyroptosis, ferroptosis, and persistent apoptosis. Key features of this period include sustained DAMP release and inflammasome activation, with caspase-1 activation leading to gasdermin D-mediated pyroptosis. Iron overload directly triggers ferroptosis or accelerates pyroptosis. Cuproptosis promotes ROS generation through the tricarboxylic acid cycle, thereby facilitating ferroptosis and pyroptosis; at the same time, cuproptosis damages mitochondria and promotes the release of cytochrome c and apoptosis-inducing factor (AIF), thereby initiating the apoptotic program. Autophagy can clear damaged organelles and inflammatory factors to exert a protective effect (pathway ②), but excessive autophagy may instead accelerate inflammation and aggravate cell death (pathway ①) (173). The late reperfusion phase results in fibroblast activation, disruption of the extracellular matrix, initiation of collagen deposition, and the establishment of a vicious cycle between persistent inflammation and cell death, ultimately leading to a further decline in cardiac function and increased myocardial stiffness.

Finally, during the remodeling phase, the predominant processes are chronic, low-grade apoptosis, persistent low-level ferroptosis, and aberrant autophagy. During this phase, signaling molecules such as TGF-*β* (released, for example, from apoptotic bodies) drive fibroblast activation and excessive collagen deposition, promoting reparative fibrosis. Ferroptosis and aberrantly activated autophagy continue to aggravate cell loss. All forms of cardiomyocyte death disrupt intercellular connections and the extracellular matrix network, impairing the homogeneity of electrical conduction. The ultimate consequences are aggravated myocardial fibrosis, a marked increase in myocardial stiffness, persistent heterogeneity of electrical conduction, a lowered threshold for arrhythmias, and an increased susceptibility to chronic heart failure.

## 5 Therapeutic strategies targeting cardiomyocyte death to ameliorate ventricular remodeling

Following myocardial infarction, irreversible cardiomyocyte injury is predominantly mediated by multiple programmed cell death pathways, including apoptosis, necrosis, pyroptosis, and ferroptosis. These interconnected processes collectively constitute the core pathological foundation for infarct progression and subsequent ventricular remodeling. Systematically delineating these mechanisms facilitates the identification of key druggable targets, thereby enabling interventions at the pathological origin to attenuate cardiomyocyte loss and ameliorate adverse remodeling.

### 5.1 Pharmacological interventions targeting individual cell death pathways

#### 5.1.1 Necrosis

The therapeutic strategy targeting necrosis primarily centers on upstream prevention and metabolic intervention, rather than precise blockade of specific signaling pathways. Necrosis is predominantly driven by severe ATP depletion, mitochondrial calcium overload, and uncontrolled opening of mPTP, ultimately leading to cell swelling and membrane rupture (174). Consequently, the main therapeutic approaches include timely reperfusion to restore blood flow and halt the ischemic process; metabolic modulators (e.g., trimetazidine) to optimize myocardial energy metabolism and delay ATP depletion; and mPTP inhibitors (e.g., cyclosporine A) to directly block mPTP opening, thereby inhibiting the final common pathway of cellular disintegration. Studies have shown that combined application of these interventions during early reperfusion significantly reduces cardiomyocyte loss mediated by passive necrosis; however, it should be noted that reperfusion itself may induce oxidative stress, partially counteracting the protective effects (175). Overall, the treatment strategy for necrosis places greater emphasis on upstream protection within the therapeutic time window rather than on molecularly targeted intervention.

#### 5.1.2 Necroptosis

Necroptosis is a form of programmed necrosis primarily driven by the RIPK1-RIPK3-MLKL axis, which is commonly activated upon stimulation by death receptor signaling such as that initiated by TNF-α (176). RIPK1 serves as an upstream signaling adaptor in this pathway, and Necrostatin-1, recognized as the first RIPK1 inhibitor (177), represents a classic experimental tool compound that effectively suppresses its kinase activity, thereby blocking necroptosis in cardiomyocytes. RIPK1 inhibitors including GSK2982772 and DNL758 have progressed into clinical trials, demonstrating potential therapeutic value in inflammatory diseases (178). RIPK3 acts as the central kinase in this cascade, playing an essential role in linking RIPK1 to MLKL. GSK’843 is a potent and high-affinity RIPK3 inhibitor capable of binding to and inhibiting its activity (179). MLKL functions as the terminal executor protein of the pathway, whose full activation culminates in plasma membrane rupture. Necrosulfonamide, as an MLKL inhibitor, prevents MLKL membrane translocation and pore formation (180), thereby directly inhibiting this inflammatory cell death program.

Within cardiomyocytes, there also exists a specific death pathway mediated by CaMKII, which can be effectively inhibited by pharmacological agents such as KN-93 or AIP (181). Furthermore, adjunctive anti-inflammatory and antioxidant therapies can be utilized to mitigate the secondary tissue damage induced by the release of necrotic cellular contents. The timely, sequential combination of the aforementioned therapeutic approaches during the early reperfusion phase is designed to synergistically reduce irreversible cardiomyocyte loss and attenuate the inflammatory response at multiple levels, ultimately aiming to ameliorate adverse ventricular remodeling and improve cardiac function.

#### 5.1.3 Apoptosis

Therapeutic strategies targeting apoptosis employ multi-level interventions centered on its core signaling pathways. At the initiation stage, intervention can be achieved by modulating the balance of the Bcl-2 protein family, for instance, using BH3 mimetics to selectively inhibit anti-apoptotic proteins and thereby relieve their blockade on pro-apoptotic proteins, or employing pharmacological agents to enhance the function of anti-apoptotic proteins such as Bcl-2 (182). Regarding the key mitochondrial events, inhibitors of the mitochondrial permeability transition pore or mitochondria-targeted antioxidants can be applied to suppress mitochondrial outer membrane permeabilization and the release of cytochrome c (183).

Targeting the common downstream executioners of apoptosis, attempts have been made to employ broad-spectrum or specific Caspase inhibitors to directly block the terminal steps of the apoptotic program; however, such strategies have not achieved success in clinical trials due to systemic side effects including a narrow therapeutic window, potential immunosuppressive effects, and impacts on normal tissue renewal. For example, the pan-caspase inhibitor emricasan advanced to Phase III trials with a favorable safety profile but ultimately failed due to insufficient efficacy in nonalcoholic steatohepatitis; GS-9450 was terminated early after inducing significant hepatotoxicity in a Phase II study (184); A systematic review has indicated that the structural conservation among caspase family members hampers the development of selective inhibitors, and together with the interference of these agents with the pleiotropic functions of caspases in normal tissue renewal and immune regulation, no caspase inhibitor has yet received regulatory approval for clinical use (185). Clinically validated indirect strategies involve the application of renin-angiotensin-aldosterone system inhibitors and *β*-blockers, which suppress upstream apoptotic signaling by reducing myocardial load and oxidative stress (186), while SGLT2 inhibitors exert anti-apoptotic effects by improving myocardial energy metabolism and endoplasmic reticulum homeostasis. Future development will focus on creating cardiac-targeted delivery systems to enhance drug specificity and reduce systemic side effects, concurrently emphasizing the temporal combination of direct and indirect intervention strategies to synergistically block the apoptotic cascade, thereby effectively reducing cardiomyocyte loss post-myocardial infarction and mitigating adverse ventricular remodeling.

#### 5.1.4 Ferroptosis

Therapeutic strategies targeting ferroptosis focus on precise interventions in its three core pathological processes: first, to address iron dyshomeostasis, iron chelators (such as deferoxamine and deferiprone) can be used to directly reduce the labile iron pool that catalyzes lipid peroxidation, and to modulate iron transporters or inhibit ferritinophagy to maintain intracellular iron homeostasis (187); second, to counteract the collapse of the antioxidant defense system, GPX4 activators or glutathione precursors can be applied to restore key phospholipid hydroperoxide scavenging capacity (188), while utilizing Nrf2 pathway activators to broadly upregulate the endogenous antioxidant defense network (189); third, to mitigate the “lipid peroxidation storm”, highly specific radical-trapping antioxidants can be employed to directly neutralize lipid radicals and interrupt chain reactions, supplemented by lipoxygenase or ACSL4 inhibitors to reduce the synthesis of peroxidation-susceptible lipids (190).

Furthermore, certain multi-target lipophilic antioxidants as well as approved cardiovascular drugs have also been found to suppress ferroptosis via indirect mechanisms. Future research aims to develop cardiac-targeted nanodelivery systems to enhance the specificity and safety of the aforementioned interventional strategies, and to combine them temporally with therapies targeting other forms of cell death, thereby synergistically blocking oxidative cardiomyocyte death, mitigating inflammatory injury, limiting infarct size, and ultimately improving ventricular remodeling and cardiac function.

#### 5.1.5 Pyroptosis

Therapeutic strategies targeting pyroptosis require multi-layered precision interventions based on its core signaling pathways: upstream, specific NLRP3 inflammasome inhibitors can directly block its ATPase activity, while ASC oligomerization inhibitors may prevent inflammasome assembly and activation (191); at the effector stage, Caspase-1 inhibitors are employed to interrupt signal transduction to downstream events; during the terminal execution phase, GSDMD inhibitors prevent pore formation by cleaved Gasdermin D, thereby directly suppressing pyroptotic cell rupture. For key inflammatory cytokines released during pyroptosis, IL-1 receptor antagonists or IL-1β neutralizing antibodies can be utilized to block generated inflammatory signals. Multi-target agents such as colchicine may also exert therapeutic effects by indirectly inhibiting NLRP3 activation (192). Future strategies will focus on developing cardiac-targeted delivery systems to enhance drug specificity, as well as implementing sequential combination therapies that integrate the aforementioned inhibitors with reperfusion therapy and blockers of other cell death pathways, aiming to synergistically disrupt the inflammatory death cascade, thereby limiting infarct size, attenuating the inflammatory storm, and ultimately improving ventricular remodeling.

#### 5.1.6 Cuproptosis

Targeted therapy for copper death-mediated post-myocardial infarction ventricular remodeling hinges on precisely restoring copper homeostasis and blocking its cytotoxic pathways, with current strategies primarily focusing on the application of copper chelators and the development of novel regulatory molecules. Highly selective copper chelators, represented by tetrathiomolybdate and triethylenetetramine, can effectively reduce bioavailable copper levels by forming stable ternary complexes (193), thereby alleviating copper ion-driven Fenton reactions, lipid peroxidation, and mitochondrial dysfunction, and have demonstrated effects in inhibiting pathological progression and improving cardiac function in models of atherosclerosis and diabetic cardiomyopathy. However, traditional chelators are limited by risks of systemic copper deficiency, interference with essential cuproprotein functions, and potential neurotoxicity; thus, research emphasis is shifting toward more specific intracellular copper transport regulators, such as small-molecule compounds targeting the copper chaperones ATOX1 and CCS, which can precisely interfere with copper delivery to specific target proteins (194), aiming to suppress pathological cuproptosis signaling while preserving normal physiological copper requirements. An ideal future therapeutic strategy should involve developing intelligent agents capable of spatiotemporally specific modulation of copper homeostasis in the ischemic myocardium, while synergistically inhibiting oxidative stress and proteotoxic stress, thereby precisely reversing cuproptosis-associated cardiomyocyte loss and fibrosis to improve ventricular remodeling outcomes.

### 5.2 Combined inhibition of multiple cell death pathways: a novel strategy for synergistic protection

Combined inhibition of multiple cell death pathways as a synergistic protective strategy is conceptually centered on employing multi-target, temporally sequenced interventions to overcome signal evasion and pathway redundancy that may occur following the blockade of a single death pathway. Preclinical studies have validated the potential of this approach, yet clinical trial evidence remains insufficient. For example, the early reperfusion combination of an NLRP3 inflammasome inhibitor (e.g., INF39) and a mitochondrial permeability transition pore inhibitor (e.g., cyclosporine A) can simultaneously block the inflammatory storm triggered by pyroptosis and the terminal execution step of classical necrosis (195). However, cyclosporine A failed to significantly improve clinical outcomes in patients with acute myocardial infarction in trials such as the CIRCUS study, partly due to its inadequate cardiac targeting and poor blood-brain barrier permeability, resulting in subtherapeutic local myocardial drug concentrations; moreover, mPTP inhibition alone cannot block cell death driven by alternative signaling pathways (e.g., the RIPK3-MLKL axis) during reperfusion (196). On this basis, sequential administration of an iron chelator (e.g., deferasirox) and a caspase inhibitor (e.g., emricasan) is employed to counter the ensuing exacerbated oxidative stress and suppress the apoptotic program. Deferasirox was shown in a phase II clinical trial of patients with myocardial infarction complicated by iron overload to reduce intramyocardial hemorrhage and infarct size, but it did not lower the incidence of major adverse cardiovascular events, suggesting that ferroptosis is only one component within the mechanistic network of reperfusion injury. Emricasan failed to improve ejection fraction in a phase II trial of chronic heart failure patients, likely because caspase inhibitors cannot block non-apoptotic death pathways (e.g., necroptosis, pyroptosis), and their limited oral bioavailability restricts efficacy in acute reperfusion injury. Concomitant use of an RIPK1-RIPK3-MLKL pathway inhibitor (e.g., GSK'963 or dabrafenib) prevents the transition from apoptosis to necroptosis. Dabrafenib (already approved for oncology) has demonstrated cardioprotective effects in animal models of myocardial ischemia-reperfusion injury, but has not yet entered cardiovascular clinical trials; its strong central nervous system penetration may lead to off-target side effects, and the potential impact of long-term MAPK pathway inhibition on cardiomyocyte homeostasis requires evaluation. Furthermore, pharmacologically maintaining unobstructed autophagic flux (e.g., using rapamycin or trimetazidine) sustains the protective function of clearing damaged organelles. Rapamycin analogs (e.g., everolimus) are used to suppress rejection in heart transplant recipients; however, their immunosuppressive effects necessitate balancing benefit against infection risk when translating this strategy to acute myocardial infarction.

Important reasons for the failure of such combination strategies include the complex cross-talk and compensatory mechanisms among different cell death pathways, whereby single- or dual-target inhibition often induces compensatory activation of alternative routes. Concurrently, preclinical studies predominantly employ young, healthy animal models, overlooking the modulatory impact of comorbidity factors such as advanced age, diabetes, and hypertension in clinical patients on signaling pathway activity. Moreover, the timing of interventions is critically important; ultra-early administration (within minutes of reperfusion) is difficult to achieve in clinical practice, and delayed administration may miss the optimal therapeutic window. Therefore, current research efforts should pivot toward screening precise biomarkers (e.g., plasma phosphorylated MLKL, Gasdermin D fragments) to identify which patient subgroups are most likely to benefit from specific combination regimens, and toward developing cardiac-targeted drug delivery systems (e.g., nanoparticle-encapsulated multi-target inhibitors) to enhance local drug efficacy.

In summary, this combination strategy targets not only pathway-specific nodes but, more importantly, shared upstream events (e.g., reactive oxygen species burst, mitochondrial dysfunction) and downstream cross-talk (e.g., activation of inflammasomes by damage-associated molecular patterns released from necrosis), thereby constructing a synergistic protective network spanning the entire process from acute injury to chronic remodeling. Nevertheless, current clinical evidence does not support immediate translational application; rigorous adaptive clinical trial designs, incorporating patient stratification and temporally sequenced dosing regimens, are required to validate the safety and efficacy of this approach. Ultimately, this strategy aims to fundamentally reduce irreversible cardiomyocyte loss, significantly dampen inflammatory responses, and steer repair processes toward a favorable direction, providing a systematic and potent solution beyond monotherapy for ameliorating ventricular remodeling.

### 5.3 Potential regulatory effects of conventional anti-remodeling agents on cell death

Conventional pharmacological agents for anti-ventricular remodeling primarily include renin-angiotensin-aldosterone system inhibitors, beta-blockers, mineralocorticoid receptor antagonists, and sodium-glucose cotransporter-2 inhibitors. These drugs act through direct or indirect mechanisms to attenuate cardiomyocyte death, suppress fibrosis, and improve hemodynamic and metabolic profiles, thereby effectively delaying or even partially reversing ventricular hypertrophy and dilation, enhancing both systolic and diastolic cardiac function, and significantly reducing the risk of heart failure hospitalization and mortality, collectively constituting the cornerstone of contemporary pharmacotherapy for heart failure.

#### 5.3.1 Renin-angiotensin-aldosterone system inhibitors

Inhibitors of the renin-angiotensin-aldosterone system primarily include angiotensin-converting enzyme inhibitors, angiotensin II receptor blockers, angiotensin receptor-neprilysin inhibitors, and mineralocorticoid receptor antagonists. These agents exert a central regulatory role in cardiomyocyte death by antagonizing angiotensin II and aldosterone signaling pathways, while also considering the inhibition of neprilysin-mediated degradation.

The cornerstone status of RAAS inhibitors is derived from multiple landmark clinical trials. The CONSENSUS study was the first to demonstrate that enalapril reduced total mortality by 40% in patients with NYHA class IV heart failure (197); subsequently, the SOLVD-Treatment trial further showed that enalapril lowered the risk of death by 16% and reduced heart failure hospitalization by 26% in patients with mild-to-moderate heart failure (198). However, the early direct renin inhibitor aliskiren failed to demonstrate clinical benefit superior to enalapril in the ATMOSPHERE trial, indicating that RAAS blockade is not unconditionally enhanced without incurring harm (199).

Angiotensin II activates NADPH oxidase through the AT1 receptor (200), triggering reactive oxygen species burst, inducing endoplasmic reticulum stress and calcium overload, thereby robustly activating the mitochondrial-dependent apoptotic pathway; its pro-inflammatory properties further exacerbate the pathological microenvironment of necroptosis and pyroptosis. Aldosterone amplifies cell death signaling by promoting oxidative stress and fibrosis (201). By inhibiting the generation or blocking the receptor of angiotensin II (as with ACEIs, ARBs, and ARNIs) or antagonizing the mineralocorticoid receptor (as with MRAs) (202), these agents systematically attenuate the aforementioned oxidative damage, calcium dyshomeostasis, and inflammatory responses, thereby significantly suppressing cardiomyocyte apoptosis, improving mitochondrial function to reduce necrotic risk, and indirectly modulating autophagic dysfunction while alleviating the pro-pyroptotic microenvironment. Ultimately, through this upstream intervention targeting multiple cell death pathways, such agents effectively mitigate irreversible cardiomyocyte loss and delay the progression of adverse ventricular remodeling.

#### 5.3.2 β-Adrenergic receptor blockers

The landmark *β*-blocker trials, MERIT-HF (metoprolol) (203), CIBIS-II (bisoprolol) (204), and COPERNICUS (carvedilol) (205), consistently demonstrated that adding a *β*-blocker to RAAS inhibitor therapy reduces all-cause mortality by approximately 34-35% and markedly lowers the risk of sudden death. Of note, bucindolol failed to gain widespread acceptance in the BEST trial due to inconsistent efficacy and racial differences, highlighting the molecular heterogeneity among different *β*-blockers (206).

As representative *β*-blockers, metoprolol and bisoprolol exert a core indirect regulatory effect on cardiomyocyte death by antagonizing the excessive stimulation of *β*1-adrenergic receptors by catecholamines (207). Sustained catecholamine activation leads to hyperphosphorylation of L-type calcium channels and ryanodine receptors via the cAMP-PKA pathway (208), triggering severe sarcoplasmic reticulum calcium leak and cytosolic calcium overload, accompanied by reactive oxygen species burst and energy depletion, thereby strongly inducing the mitochondrial-dependent apoptotic pathway and increasing the risk of necrosis. By blocking this upstream signaling, *β*-blockers effectively improve calcium handling, attenuate calcium overload and oxidative stress, and reduce myocardial oxygen consumption to alleviate the energy crisis, thereby significantly inhibiting cardiomyocyte apoptosis, reducing the susceptibility to mitochondrial permeability transition pore opening to limit necrosis, and helping to restore the physiological regulation of autophagic flux through an overall reduction in cardiac stress levels (209).During cardiac ischemia/reperfusion injury, the unique protective effects exerted by beta-blockers, particularly metoprolol, extend beyond their traditional hemodynamic regulatory functions. On the one hand, in terms of anti-inflammatory activity and neutrophil modulation, metoprolol acts directly on neutrophils in a non-classical manner that is extremely rare among beta-blockers. Through biased agonism of the *β*1-adrenergic receptor, it significantly inhibits neutrophil migration, infiltration, and inflammasome activation, thereby attenuating myocardial damage mediated by the inflammatory burst during the reperfusion phase. On the other hand, metoprolol acts at the cellular level to reduce the production of lipid peroxidation products and maintain levels of reduced glutathione, thereby effectively counteracting oxidative stress and preserving the integrity of the cardiomyocyte membrane (210–212).Their anti-inflammatory and antioxidant properties (particularly prominent in agents such as carvedilol) can indirectly suppress the activation microenvironment of pyroptosis and ferroptosis. Ultimately, through such multi-pathway upstream intervention, these agents reduce progressive cardiomyocyte loss and delay adverse ventricular remodeling.

#### 5.3.3 Mineralocorticoid receptor antagonists

The mineralocorticoid receptor antagonist (MRA) exerts a crucial inhibitory effect on cardiomyocyte death by specifically blocking the binding of aldosterone to its receptor (213). Activation of the aldosterone-MR complex potently upregulates NADPH oxidase expression and activates the NF-*κ*B pathway, thereby triggering intense oxidative stress and inflammatory responses (214), upregulating pro-apoptotic proteins while suppressing anti-apoptotic protein expression, which strongly drives the mitochondrial-dependent apoptotic pathway. By antagonizing MR, MRA systematically alleviates oxidative damage, inhibits inflammatory signaling, and blocks profibrotic gene transcription, thereby fundamentally improving the pathological microenvironment of cell death (215). This leads to a marked suppression of cardiomyocyte apoptosis, necrosis, and necroptosis, and indirectly protects cardiomyocyte survival through fibrosis reversal, ultimately working synergistically to reduce cardiomyocyte loss and delay ventricular remodeling.

The mineralocorticoid receptor antagonist trials RALES (spironolactone, NYHA class III-IV) (216) and EPHESUS (eplerenone, post-myocardial infarction heart failure) (217) reduced all-cause mortality by 30% and 15%, respectively. However, the risks of hyperkalemia and worsening renal function have limited their widespread use in clinical practice.

#### 5.3.4 Sodium-glucose cotransporter 2 inhibitors

Sodium-glucose cotransporter 2 inhibitors exert pleiotropic regulatory effects on cardiomyocyte death through unique mechanisms of “metabolic remodeling” and “stress alleviation” (218). Representative agents such as empagliflozin and dapagliflozin promote urinary glucose and sodium excretion by inhibiting renal tubular SGLT2 (219), triggering osmotic diuresis to reduce cardiac preload and inducing a simulated “physiological fasting” state that enhances myocardial utilization of efficient fuels like ketone bodies, thereby improving ATP production efficiency and reducing reactive oxygen species generation, while indirectly mitigating calcium overload risk via decreased intracellular sodium loading. These effects collectively alleviate mechanical stress, energy crisis, oxidative stress, and endoplasmic reticulum stress in cardiomyocytes, synergistically inhibiting mitochondrial-dependent apoptosis pathways, reducing necrosis risk induced by calcium overload, countering ferroptosis driven by lipid peroxidation, and contributing to the restoration of autophagic flux homeostasis (220). Ultimately, this multi-target, upstream intervention significantly attenuates cardiomyocyte loss and retards ventricular remodeling progression.

## 6 Summary and perspectives

Future interventional strategies targeting cardiomyocyte death are expected to undergo a paradigm shift from “single-target blockade” toward “network homeostatic system modulation”. This shift centers on the fundamental recognition that apoptosis, necrosis, necroptosis, pyroptosis, ferroptosis, and autophagic cell death do not occur in isolation; rather, they constitute a dynamically interconnected network under precise spatiotemporal regulation, collectively determining the pathological trajectory of post-infarct ventricular remodeling. Guided by this insight, cutting-edge research will employ single-cell spatiotemporal transcriptomics, *in vivo* high-resolution imaging, and integrated multi-omics analyses to dynamically map the “molecular landscape of cell death” across different pathological stages and myocardial regions in both *in vivo* and *in vitro* models. This approach will enable precise identification of the transition patterns governing dominant forms of cell death, from the acute ischemic phase to chronic heart failure progression, alongside their key molecular hubs. For instance, it may elucidate the critical window role of the RIPK1/RIPK3/MLKL pathway mediating the shift from apoptosis to necroptosis during the later reperfusion phase, or clarify how imbalances in mitochondrial quality control simultaneously amplify apoptosis, pyroptosis, and ferroptosis.

However, the effective implementation of the above strategies depends not only on the rational combination of multi-target agents but also on precise intervention timing and targeted delivery technologies. At present, clinical translation faces three major core limitations. First, the narrow and dynamic nature of the therapeutic window: the window of opportunity for post-ischemic mitochondrial protectors typically spans only a few to a dozen or so hours, whereas NLRP3 inflammasome activation exhibits a biphasic pattern at different time points after reperfusion; thus, intervention that is either too early or too late may attenuate efficacy or even exacerbate injury. Second, insufficient targeting specificity of drug delivery: current administration routes (systemic oral or intravenous) struggle to effectively enrich therapeutic agents in the ischemic myocardial region; iron chelators may interfere with iron metabolism in normal tissues, while the long-term effects of lysosomal activators on systemic off-target organs remain unclear. Third, the delivery dilemma posed by cellular heterogeneity: cardiomyocytes, fibroblasts, endothelial cells, and immune cells respond completely differently to the same drug, and the current lack of cell-specific delivery vehicles (e.g., cardiomyocyte-targeted adeno-associated viruses or nanoparticles) means that off-target effects may offset anticipated benefits. Therefore, successful translation of future precision therapies urgently requires the development of myocardial tissue/cell-specific delivery systems (e.g., engineered exosomes, magnetically targeted nanoparticles) and sensor technologies capable of real-time monitoring of dynamic cell death signals, in order to achieve the therapeutic goal of delivering the right drug to the right cell at the right time.

Consequently, therapeutic strategies will evolve into a “temporally sequential, combinatorial, and cell type-specific” precision medicine paradigm. During the acute ischemic phase, combination therapy with mitochondrial protective agents that inhibit mitochondrial permeability transition pore opening and specific NLRP3 inflammasome inhibitors may be employed to synergistically suppress the initial necrosis and pyroptosis storm. In the subacute repair phase, a regimen combining iron chelators and targeted antioxidants could be administered to eliminate labile iron and disrupt the vicious cycle of reactive oxygen species. Meanwhile, pharmacological activation of transcription factor EB may be used to enhance lysosomal function and restore autophagic flux. During the chronic remodeling phase, “network-balancing modulators” of regulated cell death, such as low-dose rapamycin analogs to maintain appropriate autophagic activity or the delivery of dominant-negative MLKL mutants to moderately suppress excessive necroptosis, would be implemented.

Emerging technologies will provide critical support for this vision: CRISPR-Cas9-based gene editing enables the construction of animal models that more closely mimic human disease and holds promise for developing *in vivo* editing strategies targeting key genes in specific cell death pathways; human pluripotent stem cell-derived cardiomyocytes and three-dimensional cardiac organoids will facilitate high-throughput drug screening; while nanoscale delivery systems allow spatiotemporally precise targeting of therapeutic agents to ischemic myocardial regions, thereby minimizing systemic side effects. Ultimately, the goal of this systemic strategy extends beyond merely delaying cardiomyocyte loss to actively reversing the pathological inflammation-fibrosis vicious cycle into a microenvironment conducive to tissue repair and regeneration by modulating the cell death network. For instance, designing “smart” immunomodulatory nanoparticles that clear apoptotic debris while simultaneously polarizing macrophages toward a reparative phenotype could advance cardiac repair from conceptual frameworks to clinical breakthroughs, heralding a new era in heart failure management that shifts from a passive response to cell death toward the active reprogramming of cellular fate.

## References

[1] Jang JY Lee JM Shin Y Kim YL Yu G Bae JS. Prognostic differences between persistent HFrEF and HFrecEF following acute myocardial infarction. Front Cardiovasc Med. (2025) 12:1597947. 10.3389/fcvm.2025.1597947 40747495 PMC12310739

[2] Yellon DM Hausenloy DJ. Myocardial reperfusion injury. N Engl J Med. (2007) 357(11):1121–35. 10.1056/NEJMra071667 17855673

[3] Narendran S Pereira F Ambati J. NLRP3 Inflammasome inhibition: a potential therapeutic strategy to attenuate postinfarction adverse cardiac remodeling. JACC Basic Transl Sci. (2020) 5(12):1225–7. 10.1016/j.jacbts.2020.11.004 33427823 PMC7775956

[4] Chavez-Valdez R Martin LJ Northington FJ. Programmed necrosis: a prominent mechanism of cell death following neonatal brain injury. Neurol Res Int. (2012) 2012:257563. 10.1155/2012/257563 22666585 PMC3362209

[5] Robichaux DJ Harata M Murphy E Karch J. Mitochondrial permeability transition pore-dependent necrosis. J Mol Cell Cardiol. (2023) 174:47–55. 10.1016/j.yjmcc.2022.11.003 36410526 PMC9868081

[6] Hu JJ Deng F Sun QS Xiong QM Min Y Feng SY. Time-restricted feeding protects against septic liver injury by reshaping gut microbiota and metabolite 3-hydroxybutyrate. Gut Microbes. (2025) 17(1):2486515. 10.1080/19490976.2025.2486515 40223164 PMC12005432

[7] Gao X Ma C Liang S Chen M He Y Lei W. PANoptosis: novel insight into regulated cell death and its potential role in cardiovascular diseases (review). Int J Mol Med. (2024) 54(3):74. 10.3892/ijmm.2024.5398 38963054 PMC11254103

[8] Sharma VA Frishman WH. Copper dysregulation and cardiovascular disease: a review of underlying mechanisms and therapeutic targets. Cardiol Rev. (2025). 10.1097/CRD.0000000000001051 40968409

[9] Guo L Xu CE. Integrated bioinformatics and machine learning algorithms reveal the critical cellular senescence-associated genes and immune infiltration in heart failure due to ischemic cardiomyopathy. Front Immunol. (2023) 14:1150304. 10.3389/fimmu.2023.1150304 37234159 PMC10206252

[10] Zhang M Pu D Meng F Shi G Li J. Alterations in signaling pathways and therapeutic strategies of traditional Chinese medicine in granulomatous lobular mastitis. J Inflamm Res. (2025) 18:9185–97. 10.2147/JIR.S535195 40687141 PMC12274273

[11] Natarajan V Mah T Peishi C Tan SY Chawla R Arumugam TV. Oxygen glucose deprivation induced prosurvival autophagy is insufficient to rescue endothelial function. Front Physiol. (2020) 11:533683. 10.3389/fphys.2020.533683 33041854 PMC7526687

[12] Bostan MM Stătescu C Anghel L Șerban IL Cojocaru E Sascău R. Post-myocardial infarction ventricular remodeling biomarkers—the key link between pathophysiology and clinic. Biomolecules. (2020) 10(11):1587. 10.3390/biom10111587 33238444 PMC7700609

[13] Glinton KE Ma W Lantz C Grigoryeva LS DeBerge M Liu X. Macrophage-produced VEGFC is induced by efferocytosis to ameliorate cardiac injury and inflammation. J Clin Invest. (2022) 132(9):e140685. 10.1172/JCI140685 35271504 PMC9057589

[14] Sahoo G Samal D Khandayataray P Murthy MK. A review on caspases: key regulators of biological activities and apoptosis. Mol Neurobiol. (2023) 60(10):5805–37. 10.1007/s12035-023-03433-5 37349620

[15] Wu X Li Y Zhang S Zhou X. Ferroptosis as a novel therapeutic target for cardiovascular disease. Theranostics. (2021) 11(7):3052–9. 10.7150/thno.54113 33537073 PMC7847684

[16] Chen X Tian PC Wang K Wang M Wang K. Pyroptosis: role and mechanisms in cardiovascular disease. Front Cardiovasc Med. (2022) 9:897815. 10.3389/fcvm.2022.897815 35647057 PMC9130572

[17] Xiang Q Yi X Zhu XH Wei X Jiang DS. Regulated cell death in myocardial ischemia–reperfusion injury. Trends Endocrinol Metab. (2024) 35(3):219–34. 10.1016/j.tem.2023.10.010 37981501

[18] Frangogiannis NG. Pathophysiology of myocardial infarction. Compr Physiol. (2015) 5(4):1841–75. 10.1002/cphy.c150006 26426469

[19] Krijnen PAJ. Apoptosis in myocardial ischaemia and infarction. J Clin Pathol. (2002) 55(11):801–11. 10.1136/jcp.55.11.801 12401816 PMC1769793

[20] He L Nguyen NB Ardehali R Zhou B. Heart regeneration by endogenous stem cells and cardiomyocyte proliferation: controversy, fallacy, and progress. Circulation. (2020) 142(3):275–91. 10.1161/CIRCULATIONAHA.119.045566 32687441 PMC7374760

[21] Diwan A Krenz M Syed FM Wansapura J Ren X Koesters AG. Inhibition of ischemic cardiomyocyte apoptosis through targeted ablation of Bnip3 restrains postinfarction remodeling in mice. J Clin Invest. (2007) 117(10):2825–33. 10.1172/JCI32490 17909626 PMC1994631

[22] Komai K Kawasaki NK Higa JK Matsui T. The role of ferroptosis in adverse left ventricular remodeling following acute myocardial infarction. Cells. (2022) 11(9):1399. 10.3390/cells11091399 35563704 PMC9102292

[23] Chen L Zhang LW Pan XF Liu X. Etomidate ameliorates ferroptosis and mitochondrial damage in myocardial ischemia/reperfusion injury. J Physiol Pharmacol. (2025) 76(1):10.26402/jpp.2025.1.01. 10.26402/jpp.2025.1.01 40137844

[24] Shen S Wang Z Sun H Ma L. Role of NLRP3 inflammasome in myocardial ischemia-reperfusion injury and ventricular remodeling. Med Sci Monit. (2022) 28:e934255. 10.12659/MSM.934255 35042840 PMC8790935

[25] Xu T Ding W Tariq MA Wang Y Wan Q Li M. Molecular mechanism and therapy application of necrosis during myocardial injury. J Cell Mol Med. (2018) 22(5):2547–57. 10.1111/jcmm.13575 29493109 PMC5908099

[26] Fessler EB Chibane FL Wang Z Chuang DM. Potential roles of HDAC inhibitors in mitigating ischemia-induced brain damage and facilitating endogenous regeneration and recovery. Curr Pharm Des. (2013) 19(28):5105–20. 10.2174/1381612811319280009 23448466 PMC6322545

[27] Jhelum P Karisetty BC Kumar A Chakravarty S. Implications of epigenetic mechanisms and their targets in cerebral ischemia models. Curr Neuropharmacol. (2017) 15(6):815–30. 10.2174/1570159X14666161213143907 27964703 PMC5652028

[28] Dwyer KD Snyder CA Coulombe KLK. Cardiomyocytes in hypoxia: cellular responses and implications for cell-based cardiac regenerative therapies. Bioengineering (Basel). (2025) 12(2):154. 10.3390/bioengineering12020154 40001674 PMC11851968

[29] Villa E Saso L Chichiarelli S Rojas-Solé C Pinilla-González V Prieto JC. Antioxidant cardioprotection in acute myocardial infarction: from mechanisms to therapeutic strategies. Front Biosci (Landmark Ed). (2025) 30(8):27678. 10.31083/FBL27678 40917043

[30] Zindel J Kubes P. DAMPs, PAMPs, and LAMPs in immunity and sterile inflammation. Annu Rev Pathol Mech Dis. (2020) 15(1):493–518. 10.1146/annurev-pathmechdis-012419-032847 31675482

[31] Li Z Wang Y Yuan X Xu M Wang X Liu C. Peptide-modified mesoporous silica nanoparticles for the coordinated regulation of macrophage polarization and pyroptosis in the treatment of implant-related infections. Mater Today Bio. (2025) 31:101629. 10.1016/j.mtbio.2025.101629 40124338 PMC11930442

[32] Grossman W Jones D McLaurin LP. Wall stress and patterns of hypertrophy in the human left ventricle. J Clin Invest. (1975) 56(1):56–64. 10.1172/JCI108079 124746 PMC436555

[33] Suthahar N Meijers WC Silljé HHW de Boer RA. From inflammation to fibrosis-molecular and cellular mechanisms of myocardial tissue remodelling and perspectives on differential treatment opportunities. Curr Heart Fail Rep. (2017) 14(4):235–50. 10.1007/s11897-017-0343-y 28707261 PMC5527069

[34] Mastoor Y Murphy E Roman B. Mechanisms of postischemic cardiac death and protection following myocardial injury. J Clin Invest. (2025) 135(1):e184134. 10.1172/JCI184134 39744953 PMC11684816

[35] Wang K Wang Z Ma C Yang J Xing S Song B. Mitochondria: a key regulator of programmed cell death in OP. Front Endocrinol (Lausanne). (2025) 16:1576597. 10.3389/fendo.2025.1576597 40671910 PMC12263366

[36] Aniceto N Marques V Amaral JD Serra PA Moreira R Rodrigues CMP. Harnessing protein-ligand interaction fingerprints to predict new scaffolds of RIPK1 inhibitors. Molecules. (2022) 27(15):4718. 10.3390/molecules27154718 35897894 PMC9330098

[37] Cohen L Sagi I Bigelman E Solomonov I Aloshin A Ben-Shoshan J. Cardiac remodeling secondary to chronic volume overload is attenuated by a novel MMP9/2 blocking antibody. PLoS One. (2020) 15(4):e0231202. 10.1371/journal.pone.0231202 32271823 PMC7145114

[38] Majno G Joris I. Apoptosis, oncosis, and necrosis. An overview of cell death. Am J Pathol. (1995) 146(1):3–15.PMID: 7856735 7856735 PMC1870771

[39] Kerr JFR. History of the events leading to the formulation of the apoptosis concept. Toxicology. (2002) 181–182:471–4. 10.1016/S0300-483X(02)00457-2 12505355

[40] Chaudhry GES Akim AM Sung YY Muhammad TST. Cancer and apoptosis. Methods Mol Biol. (2022) 2543:191–210. 10.1007/978-1-0716-2553-8_16 36087269

[41] Bertheloot D Latz E Franklin BS. Necroptosis, pyroptosis and apoptosis: an intricate game of cell death. Cell Mol Immunol. (2021) 18(5):1106–21. 10.1038/s41423-020-00630-3 33785842 PMC8008022

[42] Hu Z Chen H Long Y Li P Gu Y. The main sources of circulating cell-free DNA: apoptosis, necrosis and active secretion. Crit Rev Oncol Hematol. (2021) 157:103166. 10.1016/j.critrevonc.2020.103166 33254039

[43] Cheng X Ferrell JE. Apoptosis propagates through the cytoplasm as trigger waves. Science. (2018) 361(6402):607–12. 10.1126/science.aah4065 30093599 PMC6263143

[44] Kim IW Choi RY Lee JH Seo M Lee HJ Kim MA. Anticancer activity of periplanetasin-5, an antimicrobial peptide from the cockroach periplaneta Americana. J Microbiol Biotechnol. (2021) 31(10):1343–9. 10.4014/jmb.2104.04040 34409948 PMC9705916

[45] Matsumaru K Ji C Kaplowitz N. Mechanisms for sensitization to TNF-induced apoptosis by acute glutathione depletion in murine hepatocytes. Hepatology. (2003) 37(6):1425–34. 10.1053/jhep.2003.50230 12774022

[46] Kashyap D Garg VK Goel N. Intrinsic and extrinsic pathways of apoptosis: role in cancer development and prognosis. Adv Protein Chem Struct Biol. (2021) 125:73–120. 10.1016/bs.apcsb.2021.01.003 33931145

[47] Akhtar M Gallagher L Rohan S. Survivin: role in diagnosis, prognosis, and treatment of bladder cancer. Adv Anat Pathol. (2006) 13(3):122–6. 10.1097/00125480-200605000-00003 16778475

[48] Ren Z Zhang Z Ling L Liu X Wang X. Drugs for treating myocardial fibrosis. Front Pharmacol. (2023) 14:1221881. 10.3389/fphar.2023.1221881 37771726 PMC10523299

[49] Ong SB Hernández-Reséndiz S Crespo-Avilan GE Mukhametshina RT Kwek XY Cabrera-Fuentes HA. Inflammation following acute myocardial infarction: multiple players, dynamic roles, and novel therapeutic opportunities. Pharmacol Ther. (2018) 186:73–87. 10.1016/j.pharmthera.2018.01.001 29330085 PMC5981007

[50] Saedi S Tan Y Watson SE Wintergerst KA Cai L. Potential pathogenic roles of ferroptosis and cuproptosis in cadmium-induced or exacerbated cardiovascular complications in individuals with diabetes. Front Endocrinol (Lausanne). (2024) 15:1461171. 10.3389/fendo.2024.1461171 39415790 PMC11479913

[51] Steig A Miller F Shreim S Wilcox J Sykes C Whittaker D. Remote management of anaemia in patients with end-stage kidney disease using a wearable, non-invasive sensor. Clin Kidney J. (2025) 18(1):sfae375. 10.1093/ckj/sfae375 39866299 PMC11761439

[52] Kim MS Lee JH Cho HJ Cho JY Choi JO Hwang KK. KSHF Guidelines for the management of acute heart failure: part III. Specific management of acute heart failure according to the etiology and co-morbidity. Korean Circ J. (2019) 49(1):46–68. 10.4070/kcj.2018.0351 30637995 PMC6331326

[53] Ramadan M Cooper B Posnack NG. Bisphenols and phthalates: plastic chemical exposures can contribute to adverse cardiovascular health outcomes. Birth Defects Res. (2020) 112(17):1362–85. 10.1002/bdr2.1752 32691967 PMC7934580

[54] Rypdal KB Olav Melleby A Robinson EL Li J Palmero S Seifert DE. ADAMTSL3 knock-out mice develop cardiac dysfunction and dilatation with increased TGF*β* signalling after pressure overload. Commun Biol. (2022) 5(1):1392. 10.1038/s42003-022-04361-1 36539599 PMC9767913

[55] Mapuskar KA London B Zacharias ZR Houtman JCD Allen BG. Immunometabolism in the aging heart. J Am Heart Assoc. (2025) 14(1):e039216. 10.1161/JAHA.124.039216 39719411 PMC12054428

[56] Huang Y Zheng X Ming X Jiao Q Xiao W Wu Q. Recent advances in crocodilian oil research: bioactive components and potential therapeutic applications. Front Med (Lausanne). (2025) 12:1573925. 10.3389/fmed.2025.1573925 40606466 PMC12213835

[57] Di Giovanni B Gustafson D Arivalagan P Adamson MB Vishram-Nielsen J Delgado D. Elucidating associations between technetium pyrophosphate scintigraphy, echocardiography and cardiac biomarkers in transthyretin cardiac amyloidosis. Open Heart. (2025) 12(2):e002686. 10.1136/openhrt-2024-002686 40841121 PMC12374618

[58] Wang L Ge X Zhang Z Ye Y Zhou Z Li M. Identification of a ferroptosis-related long noncoding RNA prognostic signature and its predictive ability to immunotherapy in hepatocellular carcinoma. Front Genet. (2021) 12:682082. 10.3389/fgene.2021.682082 34745200 PMC8566703

[59] Shao G Qian Y Lu L Liu Y Wu T Ji G. Research progress in the role and mechanism of LPCAT3 in metabolic related diseases and cancer. J Cancer. (2022) 13(8):2430–9. 10.7150/jca.71619 35711841 PMC9174858

[60] Dixon SJ Lemberg KM Lamprecht MR Skouta R Zaitsev EM Gleason CE. Ferroptosis: an iron-dependent form of nonapoptotic cell death. Cell. (2012) 149(5):1060–72. 10.1016/j.cell.2012.03.042 22632970 PMC3367386

[61] Wu W Bu W Tan Y Wang Y. Effect of sulfasalazine on ferroptosis during intestinal injury in rats after liver transplantation. Sci Rep. (2024) 14(1):7349. 10.1038/s41598-024-58057-z 38538748 PMC10973495

[62] Yang X Liu Y Wang Z Jin Y Gu W. Ferroptosis as a new tool for tumor suppression through lipid peroxidation. Commun Biol. (2024) 7(1):1475. 10.1038/s42003-024-07180-8 39521912 PMC11550846

[63] Villalón-García I Povea-Cabello S Álvarez-Córdoba M Talaverón-Rey M Suárez-Rivero JM Suárez-Carrillo A. Vicious cycle of lipid peroxidation and iron accumulation in neurodegeneration. Neural Regen Res. (2023) 18(6):1196–202. 10.4103/1673-5374.358614 36453394 PMC9838166

[64] Parker JL Deme JC Kolokouris D Kuteyi G Biggin PC Lea SM. Molecular basis for redox control by the human cystine/glutamate antiporter system xc. Nat Commun. (2021) 12(1):7147. 10.1038/s41467-021-27414-1 34880232 PMC8654953

[65] Shimada K Stockwell BR. tRNA synthase suppression activates *de novo* cysteine synthesis to compensate for cystine and glutathione deprivation during ferroptosis. Mol Cell Oncol. (2016) 3(2):e1091059. 10.1080/23723556.2015.1091059 27308611 PMC4905397

[66] Liang D Minikes AM Jiang X. Ferroptosis at the intersection of lipid metabolism and cellular signaling. Mol Cell. (2022) 82(12):2215–27. 10.1016/j.molcel.2022.03.022 35390277 PMC9233073

[67] Li C Zhao W Geng D Jin Y Guan W. Targeting the interplay of cGAS-STING and ferroptosis by nanomedicine in the treatment of cancer. J Exp Clin Cancer Res. (2025) 44(1):249. 10.1186/s13046-025-03520-6 40846966 PMC12372235

[68] Su Y Ye L Hu C Zhang Y Liu J Shao L. Periodontitis as a promoting factor of T2D: current evidence and mechanisms. Int J Oral Sci. (2023) 15(1):25. 10.1038/s41368-023-00227-2 37321994 PMC10272210

[69] González-Domínguez Á Visiedo-García FM Domínguez-Riscart J González-Domínguez R Mateos RM Lechuga-Sancho AM. Iron metabolism in obesity and metabolic syndrome. Int J Mol Sci. (2020) 21(15):5529. 10.3390/ijms21155529 32752277 PMC7432525

[70] Na-Ek P Punsawad C. Expression of 4-hydroxynonenal (4-HNE) and heme oxygenase-1 (HO-1) in the kidneys of plasmodium berghei-infected mice. J Trop Med. (2020) 2020:8813654. 10.1155/2020/8813654 33149743 PMC7603615

[71] Rayatpour A Foolad F Heibatollahi M Khajeh K Javan M. Ferroptosis inhibition by deferiprone, attenuates myelin damage and promotes neuroprotection in demyelinated optic nerve. Sci Rep. (2022) 12(1):19630. 10.1038/s41598-022-24152-2 36385152 PMC9668997

[72] Mancuso C. Key factors which concur to the correct therapeutic evaluation of herbal products in free radical-induced diseases. Front Pharmacol. (2015) 6:86. 10.3389/fphar.2015.00086 25954201 PMC4406081

[73] Xu T Ma Y Yuan Q Hu H Hu X Qian Z. Enhanced ferroptosis by oxygen-boosted phototherapy based on a 2-in-1 nanoplatform of ferrous hemoglobin for tumor synergistic therapy. ACS Nano. (2020) 14(3):3414–25. 10.1021/acsnano.9b09426 32155051

[74] Shi J Lu Y Wei W Ma G Li C Li L. Ferroptosis: a novel pharmacological mechanism against multiple myeloma. Front Pharmacol. (2025) 16:1606804. 10.3389/fphar.2025.1606804 40735482 PMC12306488

[75] Gao J Ma N Ni S Han X. Intersection of ferroptosis and nanomaterials brings benefits to breast cancer. Cell Biol Toxicol. (2025) 41(1):119. 10.1007/s10565-025-10067-x 40691737 PMC12279609

[76] Wang J Zhu J Ren S Zhang Z Niu K Li H. The role of ferroptosis in virus infections. Front Microbiol. (2023) 14:1279655. 10.3389/fmicb.2023.1279655 38075884 PMC10706002

[77] Ros U Pedrera L Garcia-Saez AJ. Partners in crime: the interplay of proteins and membranes in regulated necrosis. Int J Mol Sci. (2020) 21(7):2412. 10.3390/ijms21072412 32244433 PMC7177786

[78] Soni P Ammal Kaidery N Sharma SM Gazaryan I Nikulin SV Hushpulian DM. A critical appraisal of ferroptosis in Alzheimer’s and Parkinson’s disease: new insights into emerging mechanisms and therapeutic targets. Front Pharmacol. (2024) 15:1390798. 10.3389/fphar.2024.1390798 39040474 PMC11260649

[79] Zhang Y Wang M Chang W. Iron dyshomeostasis and ferroptosis in Alzheimer’s disease: molecular mechanisms of cell death and novel therapeutic drugs and targets for AD. Front Pharmacol. (2022) 13:983623. 10.3389/fphar.2022.983623 36188557 PMC9523169

[80] Liu J Wang Z Lin A Zhang N. Exosomes from hypoxic pretreatment ADSCs ameliorate cardiac damage post-MI via activated circ-Stt3b/miR-15a-5p/GPX4 signaling and decreased ferroptosis. Cardiovasc Toxicol. (2024) 24(11):1215–25. 10.1007/s12012-024-09915-9 39192160 PMC11445277

[81] Jin B Zhang Z Zhang Y Yang M Wang C Xu J. Ferroptosis and myocardial ischemia-reperfusion: mechanistic insights and new therapeutic perspectives. Front Pharmacol. (2024) 15:1482986. 10.3389/fphar.2024.1482986 39411064 PMC11473306

[82] Qin S Zhu C Chen C Sheng Z Cao Y. An emerging double-edged sword role of ferroptosis in cardiovascular disease (review). Int J Mol Med. (2025) 55(1):16. 10.3892/ijmm.2024.5457 39540363 PMC11573318

[83] Zhao X Zhang M He J Li X Zhuang X. Emerging insights into ferroptosis in cholangiocarcinoma (review). Oncol Lett. (2024) 28(6):606. 10.3892/ol.2024.14739 39483963 PMC11526429

[84] Ryabov VV Maslov LN Vyshlov EV Mukhomedzyanov AV Kilin M Gusakova SV. Ferroptosis, a regulated form of cell death, as a target for the development of novel drugs preventing ischemia/reperfusion of cardiac injury, cardiomyopathy and stress-induced cardiac injury. Int J Mol Sci. (2024) 25(2):897. 10.3390/ijms25020897 38255971 PMC10815150

[85] Li Y Wang Y Guo H Wu Q Hu Y. IRF2 Contributes to myocardial infarction via regulation of GSDMD induced pyroptosis. Mol Med Rep. (2022) 25(2):40. 10.3892/mmr.2021.12556 34878155 PMC8674697

[86] Liu Q. Response by liu to letter regarding article, “cardioprotective role of tumor necrosis factor receptor-associated factor 2 by suppressing apoptosis and necroptosis.”. Circulation. (2018) 137(16):1757–8. 10.1161/CIRCULATIONAHA.117.032339 29661960 PMC5905704

[87] Guo C Deng Y Liu J Qian L. Cardiomyocyte-specific role of miR-24 in promoting cell survival. J Cell Mol Med. (2015) 19(1):103–12. 10.1111/jcmm.12393 25352422 PMC4288354

[88] Li Y Wu Z Hu J Liu G Hu H Ouyang F. Hydrogen sulfide ameliorates abdominal aorta coarctation-induced myocardial fibrosis by inhibiting pyroptosis through regulating eukaryotic translation initiation factor 2*α* phosphorylation and activating PI3 K/AKT1 pathway. Korean J Physiol Pharmacol. (2023) 27(4):345–56. 10.4196/kjpp.2023.27.4.345 37386832 PMC10316187

[89] Naryzhnaya NV Maslov LN Popov SV Mukhomezyanov AV Ryabov VV Kurbatov BK. Pyroptosis is a drug target for prevention of adverse cardiac remodeling: the crosstalk between pyroptosis, apoptosis, and autophagy. J Biomed Res. (2022) 36(6):375–89. 10.7555/JBR.36.20220123 36320147 PMC9724161

[90] Han QQ Le W. NLRP3 inflammasome-mediated neuroinflammation and related mitochondrial impairment in Parkinson’s disease. Neurosci Bull. (2023) 39(5):832–44. 10.1007/s12264-023-01023-y 36757612 PMC10169990

[91] Lin H Xiong W Fu L Yi J Yang J. Damage-associated molecular patterns (DAMPs) in diseases: implications for therapy. Mol Biomed. (2025) 6(1):60. 10.1186/s43556-025-00305-3 40877572 PMC12394712

[92] Lu X Huang H Fu X Chen C Liu H Wang H. The role of endoplasmic reticulum stress and NLRP3 inflammasome in liver disorders. Int J Mol Sci. (2022) 23(7):3528. 10.3390/ijms23073528 35408890 PMC8998408

[93] Moltrasio C Romagnuolo M Marzano AV. NLRP3 Inflammasome and NLRP3-related autoinflammatory diseases: from cryopyrin function to targeted therapies. Front Immunol. (2022) 13:1007705. 10.3389/fimmu.2022.1007705 36275641 PMC9583146

[94] Liu Y Li C Yin H Zhang X Li Y. NLRP3 Inflammasome: a potential alternative therapy target for atherosclerosis. Evid Based Complement Alternat Med. (2020) 2020:1561342. 10.1155/2020/1561342 32328119 PMC7150718

[95] Huang Y Xu W Zhou R. NLRP3 Inflammasome activation and cell death. Cell Mol Immunol. (2021) 18(9):2114–27. 10.1038/s41423-021-00740-6 34321623 PMC8429580

[96] Meng J Wang Q Wang H Shen X Qin T Zhao W. Natural products targeting NLRP3 inflammasome for metabolic dysfunction-associated fatty liver disease: the known unknowns. Chin J Nat Med. (2025) 23(9):1036–46. 10.1016/S1875-5364(25)60970-8 40976622

[97] Li CG Yan L Mai FY Shi ZJ Xu LH Jing YY. Baicalin inhibits NOD-like receptor family, pyrin containing domain 3 inflammasome activation in murine macrophages by augmenting protein kinase a signaling. Front Immunol. (2017) 8:1409. 10.3389/fimmu.2017.01409 29163487 PMC5674921

[98] de Melo ASLF Lima JLD Malta MCS Marroquim NF Moreira ÁR de Almeida Ladeia I. The role of microglia in prion diseases and possible therapeutic targets: a literature review. Prion. (2021) 15(1):191–206. 10.1080/19336896.2021.1991771 34751640 PMC8583147

[99] Du X Liu R Jiang Z Zhang C Yang Z Hu S. Chondrocyte lysates activate NLRP3 inflammasome-induced pyroptosis in synovial fibroblasts to exacerbate knee synovitis by downregulating caveolin-1. Arthritis Res Ther. (2025) 27(1):104. 10.1186/s13075-025-03573-0 40375346 PMC12083164

[100] Lin X Shi Y Zhan Y Xing Y Li Y Zhou Z. Advances of protein palmitoylation in tumor cell deaths. Cancers (Basel). (2023) 15(23):5503. 10.3390/cancers15235503 38067206 PMC10705081

[101] Vithalkar MP Pradhan S Sandra KS Bharath HB Nayak Y. Modulating NLRP3 inflammasomes in idiopathic pulmonary fibrosis: a comprehensive review on flavonoid-based interventions. Cell Biochem Biophys. (2025) 83(3):2669–701. 10.1007/s12013-025-01696-4 39966334 PMC12414096

[102] Lv H He Y Wu J Zhen L Zheng Y. Chronic cold stress-induced myocardial injury: effects on oxidative stress, inflammation and pyroptosis. J Vet Sci. (2023) 24(1):e2. 10.4142/jvs.22185 36726274 PMC9899938

[103] Guo H Callaway JB Ting JPY. Inflammasomes: mechanism of action, role in disease, and therapeutics. Nat Med. (2015) 21(7):677–87. 10.1038/nm.3893 26121197 PMC4519035

[104] Wu C Lu W Zhang Y Zhang G Shi X Hisada Y. Inflammasome activation triggers blood clotting and host death through pyroptosis. Immunity. (2019) 50(6):1401–1411.e4. 10.1016/j.immuni.2019.04.003 31076358 PMC6791531

[105] Li H Yang DH Zhang Y Zheng F Gao F Sun J. Geniposide suppresses NLRP3 inflammasome-mediated pyroptosis via the AMPK signaling pathway to mitigate myocardial ischemia/reperfusion injury. Chin Med. (2022) 17(1):73. 10.1186/s13020-022-00616-5 35715805 PMC9205109

[106] El Kazzi M Rayner BS Chami B Dennis JM Thomas SR Witting PK. Neutrophil-mediated cardiac damage after acute myocardial infarction: significance of defining a new target cell type for developing cardioprotective drugs. Antioxid Redox Signal. (2020) 33(10):689–712. 10.1089/ars.2019.7928 32517486 PMC7475094

[107] Ma Y. Role of neutrophils in cardiac injury and repair following myocardial infarction. Cells. (2021) 10(7):1676. 10.3390/cells10071676 34359844 PMC8305164

[108] Mohammed ZA Patni MM Qassim MM Naji NM Al Abid IK Kharoufeh A. A hidden pathway to a major concern: the role of pyroptosis in inducing myocardial infarction reperfusion injury and emerging therapeutic targets. Cureus. (2025) 17(12):e98370. 10.7759/cureus.98370 41487823 PMC12758088

[109] Chang X Zhang T Wang J Liu Y Yan P Meng Q. SIRT5-related Desuccinylation modification contributes to quercetin-induced protection against heart failure and high-glucose-prompted cardiomyocytes injured through regulation of mitochondrial quality surveillance. Oxid Med Cell Longev. (2021) 2021:5876841. 10.1155/2021/5876841 34603599 PMC8486530

[110] Hu Y Reggiori F. Molecular regulation of autophagosome formation. Biochem Soc Trans. (2022) 50(1):55–69. 10.1042/BST20210819 35076688 PMC9022990

[111] Yi S Qi X Luo F Wang D Feng Z Ma L. TFIIB-related factor 2 inhibits lung squamous carcinoma cell apoptosis through SLC8A3-mediated mitochondrial homeostasis. Cell Death Dis. (2025) 16(1):491. 10.1038/s41419-025-07813-8 40610422 PMC12229314

[112] Arden C. A role for glucagon-like peptide-1 in the regulation of *β*-cell autophagy. Peptides. (2018) 100:85–93. 10.1016/j.peptides.2017.12.002 29412836

[113] Bieber A Capitanio C Erdmann PS Fiedler F Beck F Lee CW. *In situ* structural analysis reveals membrane shape transitions during autophagosome formation. Proc Natl Acad Sci U S A. (2022) 119(39):e2209823119. 10.1073/pnas.2209823119 36122245 PMC9522377

[114] Forte M D’Ambrosio L Schiattarella GG Salerno N Perrone MA Loffredo FS. Mitophagy modulation for the treatment of cardiovascular diseases. Eur J Clin Invest. (2024) 54(8):e14199. 10.1111/eci.14199 38530070

[115] Galati S Boni C Gerra MC Lazzaretti M Buschini A. Autophagy: a player in response to oxidative stress and DNA damage. Oxid Med Cell Longev. (2019) 2019:5692958. 10.1155/2019/5692958 31467633 PMC6701339

[116] Yang YY Gao ZX Mao ZH Liu DW Liu ZS Wu P. Identification of ULK1 as a novel mitophagy-related gene in diabetic nephropathy. Front Endocrinol (Lausanne). (2022) 13:1079465. 10.3389/fendo.2022.1079465 36743936 PMC9889542

[117] Li L Xi R Gao B Zeng Y Ma Q Gong T. Research progress of autophagy in heart failure. Am J Transl Res. (2024) 16(5):1991–2000. 10.62347/OBXQ9477 38883358 PMC11170578

[118] Chen L Yang L Li Y Liu T Yang B Liu L. Autophagy and inflammation: regulatory roles in viral infections. Biomolecules. (2023) 13(10):1454. 10.3390/biom13101454 37892135 PMC10604974

[119] Wang C Zhu X Chen R Zhang X Lian N. Upregulation of UBR1 m6A methylation by METTL14 inhibits autophagy in spinal cord injury. eNeuro. (2023) 10(6): ENEURO.0338-22.2023. 10.1523/ENEURO.0338-22.2023 PMC10241380 37094938

[120] Ding X Zhu C Wang W Li M Ma C Gao B. SIRT1 Is a regulator of autophagy: implications for the progression and treatment of myocardial ischemia-reperfusion. Pharmacol Res. (2024) 199:106957. 10.1016/j.phrs.2023.106957 37820856

[121] Fassio A Falace A Esposito A Aprile D Guerrini R Benfenati F. Emerging role of the autophagy/lysosomal degradative pathway in neurodevelopmental disorders with epilepsy. Front Cell Neurosci. (2020) 14:39. 10.3389/fncel.2020.00039 32231521 PMC7082311

[122] Wang Y Wang X Qian Y Sun M Yang H Su L. Naringenin attenuates slow-transit constipation by regulating the AMPK/mTOR/ULK1 signalling pathway: *in vivo* and *in vitro* studies. Front Pharmacol. (2025) 16:1550458. 10.3389/fphar.2025.1550458 40599805 PMC12209368

[123] Wei Z Hu X Wu Y Zhou L Zhao M Lin Q. Molecular mechanisms underlying initiation and activation of autophagy. Biomolecules. (2024) 14(12):1517. 10.3390/biom14121517 39766224 PMC11673044

[124] Ghosh AK Mau T O’Brien M Yung R. Novel role of autophagy-associated Pik3c3 gene in gonadal white adipose tissue browning in aged C57/Bl6 male mice. Aging (Albany NY). (2018) 10(4):764–74. 10.18632/aging.101426 29695642 PMC5940123

[125] Herzig S Shaw RJ. AMPK: guardian of metabolism and mitochondrial homeostasis. Nat Rev Mol Cell Biol. (2018) 19(2):121–35. 10.1038/nrm.2017.95 28974774 PMC5780224

[126] Zheng Q Zheng X Zhang L Luo H Qian L Fu X. The neuron-specific protein TMEM59L mediates oxidative stress-induced cell death. Mol Neurobiol. (2017) 54(6):4189–200. 10.1007/s12035-016-9997-9 27324899 PMC5288309

[127] Lapaquette P Ducreux A Basmaciyan L Paradis T Bon F Bataille A. Membrane protective role of autophagic machinery during infection of epithelial cells by candida albicans. Gut Microbes. (2022) 14(1):2004798. 10.1080/19490976.2021.2004798 35086419 PMC8803057

[128] Park S Choi J Biering SB Dominici E Williams LE Hwang S. Targeting by AutophaGy proteins (TAG): targeting of IFNG-inducible GTPases to membranes by the LC3 conjugation system of autophagy. Autophagy. (2016) 12(7):1153–67. 10.1080/15548627.2016.1178447 27172324 PMC4990996

[129] Ghosh R Pattison JS. Macroautophagy and chaperone-mediated autophagy in heart failure: the known and the unknown. Oxid Med Cell Longev. (2018) 2018:8602041. 10.1155/2018/8602041 29576856 PMC5822756

[130] Zhang J Jiang N Du C Guo H Meng R Hou X. HSF4 Transcriptionally activates autophagy by regulating ATG9a during lens terminal differentiation. Invest Ophthalmol Vis Sci. (2023) 64(7):5. 10.1167/iovs.64.7.5 PMC10243497 37266953

[131] Li Y Liu R Wu J Li X. Self-eating: friend or foe? The emerging role of autophagy in fibrotic diseases. Theranostics. (2020) 10(18):7993–8017. 10.7150/thno.47826 32724454 PMC7381749

[132] Yong YY Zhang L Hu YJ Wu JM Yan L Pan YR. Targeting autophagy regulation in NLRP3 inflammasome-mediated lung inflammation in COVID-19. Clin Immunol. (2022) 244:109093. 10.1016/j.clim.2022.109093 35944881 PMC9356669

[133] Han XJ Hu YY Yang ZJ Jiang LP Shi SL Li YR. Amyloid *β*-42 induces neuronal apoptosis by targeting mitochondria. Mol Med Rep. (2017) 16(4):4521–8. 10.3892/mmr.2017.7203 28849115 PMC5647099

[134] Guo Z Tian Y Liu N Chen Y Chen X Yuan G. Mitochondrial stress as a central player in the pathogenesis of hypoxia-related myocardial dysfunction: new insights. Int J Med Sci. (2024) 21(13):2502–9. 10.7150/ijms.99359 39439461 PMC11492880

[135] Trease AJ Totusek S Lichter EZ Stauch KL Fox HS. Mitochondrial DNA instability supersedes parkin mutations in driving mitochondrial proteomic alterations and functional deficits in polg mutator mice. Int J Mol Sci. (2024) 25(12):6441. 10.3390/ijms25126441 38928146 PMC11203920

[136] Xu W Dong L Dai J Zhong L Ouyang X Li J. The interconnective role of the UPS and autophagy in the quality control of cancer mitochondria. Cell Mol Life Sci. (2025) 82(1):42. 10.1007/s00018-024-05556-x 39800773 PMC11725563

[137] Han H Hu S Hu Y Liu D Zhou J Liu X. Mitophagy in ototoxicity. Front Cell Neurosci. (2023) 17:1140916. 10.3389/fncel.2023.1140916 36909283 PMC9995710

[138] Chu YD Chen WT Lin WR Lai MW Yeh CT. Mitochondrial echoes in the bloodstream: decoding ccf-mtDNA for the early detection and prognosis of hepatocellular carcinoma. Cell Biosci. (2025) 15(1):118. 10.1186/s13578-025-01456-0 40796910 PMC12344845

[139] Liu C He W Zhang J. Exercise regulates mitophagy to alleviate parkinsonian neurodegeneration. Front Aging Neurosci. (2025) 17:1678460. 10.3389/fnagi.2025.1678460 41356234 PMC12679300

[140] Zhang X Shao S Li Q Wang Y Kong M Zhang C. Roles of autophagy, mitophagy, and mitochondria in left ventricular remodeling after myocardial infarction. Rev Cardiovasc Med. (2025) 26(3):28195. 10.31083/RCM28195 40160572 PMC11951495

[141] Ho HJ Shirakawa H. Oxidative stress and mitochondrial dysfunction in chronic kidney disease. Cells. (2022) 12(1):88. 10.3390/cells12010088 36611880 PMC9818928

[142] Bai R Zhao Z Han X Shang M Liu G Xu F. Therapeutic potential of ginsenosides in anthracycline-induced cardiotoxicity. Molecules. (2025) 30(12):2527. doi: 10.3390/molecules30122527 40572494 PMC12196387

[143] Fan C Tang X Ye M Zhu G Dai Y Yao Z. Qi-li-qiang-xin alleviates isoproterenol-induced myocardial injury by inhibiting excessive autophagy via activating AKT/mTOR pathway. Front Pharmacol. (2019) 10:1329. 10.3389/fphar.2019.01329 31780944 PMC6861302

[144] Sun Z Wang X Pang X. Potential of polydatin against ischemia-reperfusion injury: new insights from pharmacological-pathological mechanism associations. Drug Des Devel Ther. (2025) 19:1585–94. 10.2147/DDDT.S508851 40066082 PMC11892733

[145] Xu J Minobe E Kameyama M. Ca2 + dyshomeostasis links risk factors to neurodegeneration in Parkinson’s disease. Front Cell Neurosci. (2022) 16:867385. 10.3389/fncel.2022.867385 35496903 PMC9050104

[146] Chen Y Zhu X Ye F Wang H Wan X Zhang T. Malondialdehyde-modified photoreceptor outer segments promote choroidal neovascularization in mice. Transl Vis Sci Technol. (2022) 11(1):12. 10.1167/tvst.11.1.12 PMC8762676 35015060

[147] Abdellatif M Vasques-Nóvoa F Trummer-Herbst V Durand S Koser F Islam M. Autophagy is required for the therapeutic effects of the NAD+ precursor nicotinamide in obesity-related heart failure with preserved ejection fraction. Eur Heart J. (2025) 46(19):1863–6. 10.1093/eurheartj/ehaf062 39995248 PMC12075933

[148] Zhu H Tannous P Johnstone JL Kong Y Shelton JM Richardson JA. Cardiac autophagy is a maladaptive response to hemodynamic stress. J Clin Invest. (2007) 117(7):1782–93. 10.1172/JCI27523 17607355 PMC1890995

[149] Tsvetkov P Coy S Petrova B Dreishpoon M Verma A Abdusamad M. Copper induces cell death by targeting lipoylated TCA cycle proteins. Science. (2022) 375(6586):1254–61. 10.1126/science.abf0529 35298263 PMC9273333

[150] Chen L Min J Wang F. Copper homeostasis and cuproptosis in health and disease. Signal Transduct Target Ther. (2022) 7(1):378. 10.1038/s41392-022-01229-y 36414625 PMC9681860

[151] Zhou N Wei S Sun T Xie S Liu J Li W. Recent progress in the role of endogenous metal ions in doxorubicin-induced cardiotoxicity. Front Pharmacol. (2023) 14:1292088. 10.3389/fphar.2023.1292088 38143497 PMC10748411

[152] Jacobs A Renaudin G Charbonnel N Nedelec JM Forestier C Descamps S. Copper-doped biphasic calcium phosphate powders: dopant release, cytotoxicity and antibacterial properties. Materials (Basel). (2021) 14(9):2393. 10.3390/ma14092393 34064435 PMC8124198

[153] Chen H Li D Zhang H Zhang M Lin Y He H. Mechanisms of copper metabolism and cuproptosis: implications for liver diseases. Front Immunol. (2025) 16:1633711. 10.3389/fimmu.2025.1633711 40808953 PMC12343240

[154] Ke D Zhang Z Liu J Chen P Li J Sun X. Ferroptosis, necroptosis and cuproptosis: novel forms of regulated cell death in diabetic cardiomyopathy. Front Cardiovasc Med. (2023) 10:1135723. 10.3389/fcvm.2023.1135723 36970345 PMC10036800

[155] Lu X Chen X Lin C Yi Y Zhao S Zhu B. Elesclomol loaded copper oxide nanoplatform triggers cuproptosis to enhance antitumor immunotherapy. Adv Sci (Weinh). (2024) 11(18):e2309984. 10.1002/advs.202309984 38430531 PMC11095170

[156] Zhang Y Sun W Meng X Yang X Su L Huang P. Efficient copper ion transport triggers *in situ* photothermia and cuproptosis for boosting colon cancer immunotherapy. Biomaterials. (2026) 327:123759. 10.1016/j.biomaterials.2025.123759 41045759

[157] Wang S Wan L Zhang M Yan D Li F. New targets for immune inflammatory response in rheumatoid arthritis: focus on the potential significance of N6-methyladenosine, ferroptosis and cuproptosis. J Inflamm Res. (2025) 18:8085–106. 10.2147/JIR.S526096 40551986 PMC12184783

[158] Wang C Ja Y Liu T Lin K Huang L Ren C. Metal-related cell death and its application in pancreatic cancer. Expert Rev Mol Med. (2025) 27:e37. 10.1017/erm.2025.10022 41102973 PMC12671914

[159] Zhao Z Ma Y Liu Y Chen Z Zheng J. A cuproptosis-based prognostic model for predicting survival in low-grade glioma. Aging (Albany NY). (2024) 16(10):8697–716. 10.18632/aging.205834 38738989 PMC11164498

[160] Luo L Hu X Huang A Liu X Wang L Du T. A noval established cuproptosis-associated LncRNA signature for prognosis prediction in primary hepatic carcinoma. Evid Based Complement Alternat Med. (2022) 2022:2075638. 10.1155/2022/2075638 36159561 PMC9499762

[161] Nong J Lu G Huang Y Liu J Chen L Pan H. Identification of cuproptosis-related subtypes, characterization of immune microenvironment infiltration, and development of a prognosis model for osteoarthritis. Front Immunol. (2023) 14:1178794. 10.3389/fimmu.2023.1178794 37809099 PMC10551149

[162] Jiang D Zhuang L Koong AC Gan B. Cuproptosis in cancer: from molecular mechanisms to therapeutic intervention. Trends Cancer. (2026) 12(3):275–86. 10.1016/j.trecan.2025.12.002 41419376

[163] Gallo A Giral P Rosenbaum D Mattina A Kilinc A Giron A. Myocardial fibrosis assessed by magnetic resonance imaging in asymptomatic heterozygous familial hypercholesterolemia: the cholcoeur study. EBioMedicine. (2021) 74:103735. 10.1016/j.ebiom.2021.103735 34864619 PMC8646177

[164] Mrozińska Z Kudzin MH Ponczek MB Kaczmarek A Król P Lisiak-Kucińska A. Biochemical approach to poly(lactide)-copper composite-impact on blood coagulation processes. Materials (Basel). (2024) 17(3):608. 10.3390/ma17030608 38591465 PMC10856769

[165] Li P Li Y Meng Q Wang J Wang K Yang S. Copper dyshomeostasis and cardiovascular disease: molecular mechanisms and new strategies for targeted intervention with cuproptosis (review). Int J Mol Med. (2026) 57(1):19. 10.3892/ijmm.2025.5690 41235671 PMC12634067

[166] Wang Y Wen T Mao F Yang S Zhang Q Fu X. Engineering copper and copper-based materials for a post-antibiotic era. Front Bioeng Biotechnol. (2025) 13:1644362. 10.3389/fbioe.2025.1644362 40843444 PMC12364917

[167] Kober KI Cano A Géraud C Sipilä K Mobasseri SA Philippeos C. Loxl2 is dispensable for dermal development, homeostasis and tumour stroma formation. PLoS One. (2018) 13(6):e0199679. 10.1371/journal.pone.0199679 29953488 PMC6023175

[168] Jomova K Alomar SY Valko R Nepovimova E Kuca K Valko M. The role of redox-active iron, copper, manganese, and redox-inactive zinc in toxicity, oxidative stress, and human diseases. EXCLI J. (2025) 24:880–954. 10.17179/excli2025-8449 40933952 PMC12419454

[169] Guo Z Liu Y Chen D Sun Y Li D Meng Y. Targeting regulated cell death: apoptosis, necroptosis, pyroptosis, ferroptosis, and cuproptosis in anticancer immunity. J Transl Int Med. (2025) 13(1):10–32. 10.1515/jtim-2025-0004 40115032 PMC11921819

[170] Pang Q Meng X Zhou Z You L Yuan J Feng Q. Temporal regulation of genetic programs governing multiple cell death during myocardial ischemia-reperfusion injury. Front Genet. (2025) 16:1632867. 10.3389/fgene.2025.1632867 40979591 PMC12446000

[171] Qin D Modanwal R Ghazi M Corbalan JJ Jia XF Axelrod JL. Reformulation of the necroptosis pathway in reperfused myocardial infarction. Circulation. (2025) 152(7):486–8. 10.1161/CIRCULATIONAHA.124.073023 40825061 PMC12363653

[172] Li J Zhang H Dai M Huang Y. 15-lipoxygenase Blockade switches off pan-organ ischaemia-reperfusion injury by inhibiting pyroptosis. Mol Biomed. (2025) 6(1):77. 10.1186/s43556-025-00325-z 41068428 PMC12511505

[173] Qi L Yi J Ji Y Jiang H Sun J Shen Y. Network of cell death in myocardial ischemia-reperfusion injury: mechanisms and targeted therapeutic strategies. Biochem Pharmacol. (2026) 250(Pt 2):117999. 10.1016/j.bcp.2026.117999 42035955

[174] Shi S Wang L van der Laan LJW Pan Q Verstegen MMA. Mitochondrial dysfunction and oxidative stress in liver transplantation and underlying diseases: new insights and therapeutics. Transplantation. (2021) 105(11):2362–73. 10.1097/TP.0000000000003691 33577251 PMC9005104

[175] Hanna A Frangogiannis NG. The role of the TGF-*β* superfamily in myocardial infarction. Front Cardiovasc Med. (2019) 6:140. 10.3389/fcvm.2019.00140 31620450 PMC6760019

[176] Wang L Chang X Feng J Yu J Chen G. TRADD Mediates RIPK1-independent necroptosis induced by tumor necrosis factor. Front Cell Dev Biol. (2019) 7:393. 10.3389/fcell.2019.00393 32039207 PMC6987388

[177] Scarpellini C Valembois S Goossens K Vadi M Lanthier C Klejborowska G. From PERK to RIPK1: design, synthesis and evaluation of novel potent and selective necroptosis inhibitors. Front Chem. (2023) 11:1160164. 10.3389/fchem.2023.1160164 37090247 PMC10119423

[178] Vissers MFJM Heuberger JAAC Groeneveld GJ Oude Nijhuis J De Deyn PP Hadi S. Safety, pharmacokinetics and target engagement of novel RIPK1 inhibitor SAR443060 (DNL747) for neurodegenerative disorders: randomized, placebo-controlled, double-blind phase I/ib studies in healthy subjects and patients. Clin Transl Sci. (2022) 15(8):2010–23. 10.1111/cts.13317 35649245 PMC9372423

[179] Zhou Y Xiang Y Liu S Li C Dong J Kong X. RIPK3 Signaling and its role in regulated cell death and diseases. Cell Death Discov. (2024) 10(1):200. 10.1038/s41420-024-01957-w 38684668 PMC11059363

[180] Gautheron J Gores GJ Rodrigues CMP. Lytic cell death in metabolic liver disease. J Hepatol. (2020) 73(2):394–408. 10.1016/j.jhep.2020.04.001 32298766 PMC7371520

[181] Zhu W Woo AYH Yang D Cheng H Crow MT Xiao RP. Activation of CaMKIIdeltaC is a common intermediate of diverse death stimuli-induced heart muscle cell apoptosis. J Biol Chem. (2007) 282(14):10833–9. 10.1074/jbc.M611507200 17296607

[182] Saleh T Carpenter VJ Tyutyunyk-Massey L Murray G Leverson JD Souers AJ. Clearance of therapy-induced senescent tumor cells by the senolytic ABT-263 via interference with BCL-XL -BAX interaction. Mol Oncol. (2020) 14(10):2504–19. 10.1002/1878-0261.12761 32652830 PMC7530780

[183] Calvo-Rodriguez M Kharitonova EK Snyder AC Hou SS Sanchez-Mico MV Das S. Real-time imaging of mitochondrial redox reveals increased mitochondrial oxidative stress associated with amyloid *β* aggregates *in vivo* in a mouse model of Alzheimer’s disease. Mol Neurodegener. (2024) 19(1):6. 10.1186/s13024-024-00702-2 38238819 PMC10797952

[184] Ratziu V Sheikh MY Sanyal AJ Lim JK Conjeevaram H Chalasani N. A phase 2, randomized, double-blind, placebo-controlled study of GS-9450 in subjects with nonalcoholic steatohepatitis. Hepatology. (2012) 55(2):419–28. 10.1002/hep.24747 22006541 PMC3779694

[185] Dhani S Zhao Y Zhivotovsky B. A long way to go: caspase inhibitors in clinical use. Cell Death Dis. (2021) 12(10):949. 10.1038/s41419-021-04240-3 34654807 PMC8519909

[186] Udell JA Bahit MC Campbell P Butler J Chopra VK Bayés-Genís A. Prevention of heart failure after acute myocardial infarction. Lancet. (2025) 406(10508):1154–70. 10.1016/S0140-6736(25)01394-7 40889512

[187] Su LJ Zhang JH Gomez H Murugan R Hong X Xu D. Reactive oxygen species-induced lipid peroxidation in apoptosis, autophagy, and ferroptosis. Oxid Med Cell Longev. (2019) 2019:5080843. 10.1155/2019/5080843 31737171 PMC6815535

[188] Fan B Chen G Huang S Li Y Nabil ZUH Yang Z. Summary of the mechanism of ferroptosis regulated by m6A modification in cancer progression. Front Cell Dev Biol. (2025) 13:1507171. 10.3389/fcell.2025.1507171 40271153 PMC12014555

[189] Cano-Gómez CI Alonso-Castro AJ Carranza-Alvarez C Wong-Paz JE. Advancements in litchi chinensis peel processing: a scientific review of drying, extraction, and isolation of its bioactive compounds. Foods. (2024) 13(10):1461. 10.3390/foods13101461 38790761 PMC11119950

[190] Andritoiu CV Ochiuz L Andritoiu V Popa M. Effect of apitherapy formulations against carbon tetrachloride-induced toxicity in wistar rats after three weeks of treatment. Molecules. (2014) 19(9):13374–91. 10.3390/molecules190913374 25178061 PMC6270670

[191] Lee S Ye Q Yang H Lee S Kim Y Lee N. Aiouea padiformis extract exhibits anti-inflammatory effects by inhibiting the ATPase activity of NLRP3. Sci Rep. (2024) 14(1):5237. 10.1038/s41598-024-55651-z 38433281 PMC10909851

[192] Wu D Zhang Z Jiang X Du Y Zhang S Yang XD. Inflammasome meets centrosome: understanding the emerging role of centrosome in controlling inflammasome activation. Front Immunol. (2022) 13:826106. 10.3389/fimmu.2022.826106 35281071 PMC8907152

[193] Wei H Zhang WJ McMillen TS Leboeuf RC Frei B. Copper chelation by tetrathiomolybdate inhibits vascular inflammation and atherosclerotic lesion development in apolipoprotein E-deficient mice. Atherosclerosis. (2012) 223(2):306–13. 10.1016/j.atherosclerosis.2012.06.013 22770994 PMC3417757

[194] Yang Y Feng Q Luan Y Liu H Jiao Y Hao H. Exploring cuproptosis as a mechanism and potential intervention target in cardiovascular diseases. Front Pharmacol. (2023) 14:1229297. 10.3389/fphar.2023.1229297 37637426 PMC10450925

[195] Cung TT Morel O Cayla G Rioufol G Garcia-Dorado D Angoulvant D. Cyclosporine before PCI in patients with acute myocardial infarction. N Engl J Med. (2015) 373(11):1021–31. 10.1056/NEJMoa1505489 26321103

[196] Mewton N Cung TT Morel O Cayla G Bonnefoy-Cudraz E Rioufol G. Rationale and design of the cyclosporine to ImpRove clinical oUtcome in ST-elevation myocardial infarction patients (the CIRCUS trial). Am Heart J. (2015) 169(6):758–766.e6. 10.1016/j.ahj.2015.02.020 26027612

[197] Swedberg K Kjekshus J Snapinn S. Long-term survival in severe heart failure in patients treated with enalapril. Ten year follow-up of CONSENSUS I. Eur Heart J. (1999) 20(2):136–9. 10.1053/euhj.1998.1098 10099910

[198] Bowling CB Sanders PW Allman RM Rogers WJ Patel K Aban IB. Effects of enalapril in systolic heart failure patients with and without chronic kidney disease: insights from the SOLVD treatment trial. Int J Cardiol. (2013) 167(1):151–6. 10.1016/j.ijcard.2011.12.056 22257685 PMC3395757

[199] Kristensen SL Mogensen UM Tarnesby G Gimpelewicz CR Ali MA Shao Q. Aliskiren alone or in combination with enalapril vs. Enalapril among patients with chronic heart failure with and without diabetes: a subgroup analysis from the ATMOSPHERE trial. Eur J Heart Fail. (2018) 20(1):136–47. 10.1002/ejhf.896 28948656

[200] Chen M Zhu W Chen Y Shang J Wang W Yan X. Aloe-emodin ameliorates chronic kidney disease fibrosis by inhibiting PI3K-mediated signaling pathway. Eur J Histochem. (2025) 69(3):4228. 10.4081/ejh.2025.4228 40832995 PMC12406115

[201] Wang W Liu W Tang Y Sun F He H Yan Z. Clinical value of sublingual microcirculatory dysfunction for screening for primary aldosteronism in hypertensive patients. Front Endocrinol (Lausanne). (2025) 16:1561503. 10.3389/fendo.2025.1561503 40747303 PMC12310458

[202] Lund LH Crespo-Leiro MG Laroche C Zaliaduonyte D Saad AM Fonseca C. Heart failure in Europe: guideline-directed medical therapy use and decision making in chronic and acute, pre-existing and *de novo*, heart failure with reduced, mildly reduced, and preserved ejection fraction - the ESC EORP heart failure III registry. Eur J Heart Fail. (2024) 26(12):2487–501. 10.1002/ejhf.3445 39257278 PMC11683873

[203] Goldstein S Fagerberg B Hjalmarson A Kjekshus J Waagstein F Wedel H. Metoprolol controlled release/extended release in patients with severe heart failure: analysis of the experience in the MERIT-HF study. J Am Coll Cardiol. (2001) 38(4):932–8. 10.1016/s0735-1097(01)01516-9 11583861

[204] Lechat P Hulot JS Escolano S Mallet A Leizorovicz A Werhlen-Grandjean M. Heart rate and cardiac rhythm relationships with bisoprolol benefit in chronic heart failure in CIBIS II trial. Circulation. (2001) 103(10):1428–33. 10.1161/01.cir.103.10.1428 11245648

[205] Packer M Fowler MB Roecker EB Coats AJS Katus HA Krum H. Effect of carvedilol on the morbidity of patients with severe chronic heart failure: results of the carvedilol prospective randomized cumulative survival (COPERNICUS) study. Circulation. (2002) 106(17):2194–9. 10.1161/01.cir.0000035653.72855.bf 12390947

[206] Smart NA Kwok N Holland DJ Jayasighe R Giallauria F. Bucindolol: a pharmacogenomic perspective on its use in chronic heart failure. Clin Med Insights Cardiol. (2011) 5:55–66. 10.4137/CMC.S4309 21792345 PMC3140276

[207] Chen XJ Liu SY Li SM Feng JK Hu Y Cheng XZ. The recent advance and prospect of natural source compounds for the treatment of heart failure. Heliyon. (2024) 10(5):e27110. 10.1016/j.heliyon.2024.e27110 38444481 PMC10912389

[208] Cairns M Marais E Joseph D Essop MF. The role of chronic stress in the pathogenesis of ischemic heart disease in women. Compr Physiol. (2025) 15(1):e70000. 10.1002/cph4.70000 39903543 PMC11793136

[209] Ruiz-Meana M Bou-Teen D Ferdinandy P Gyongyosi M Pesce M Perrino C. Cardiomyocyte ageing and cardioprotection: consensus document from the ESC working groups cell biology of the heart and myocardial function. Cardiovasc Res. (2020) 116(11):1835–49. 10.1093/cvr/cvaa132 32384145

[210] Ibanez B Macaya C Sánchez-Brunete V Pizarro G Fernández-Friera L Mateos A. Effect of early metoprolol on infarct size in ST-segment-elevation myocardial infarction patients undergoing primary percutaneous coronary intervention: the effect of metoprolol in cardioprotection during an acute myocardial infarction (METOCARD-CNIC) trial. Circulation. (2013) 128(14):1495–503. 10.1161/CIRCULATIONAHA.113.003653 24002794

[211] García-Prieto J Villena-Gutiérrez R Gómez M Bernardo E Pun-García A García-Lunar I. Neutrophil stunning by metoprolol reduces infarct size. Nat Commun. (2017) 8:14780. 10.1038/ncomms14780 28416795 PMC5399300

[212] Clemente-Moragón A Gómez M Villena-Gutiérrez R Lalama DV García-Prieto J Martínez F. Metoprolol exerts a non-class effect against ischaemia-reperfusion injury by abrogating exacerbated inflammation. Eur Heart J. (2020) 41(46):4425–40. 10.1093/eurheartj/ehaa733 33026079 PMC7752252

[213] Loan Le TY Mardini M Howell VM Funder JW Ashton AW Mihailidou AS. Low-dose spironolactone prevents apoptosis repressor with caspase recruitment domain degradation during myocardial infarction. Hypertension. (2012) 59(6):1164–9. 10.1161/HYPERTENSIONAHA.111.190488 22508833

[214] Zhou M Lv J Chen X Shi Y Chao G Zhang S. From gut to liver: exploring the crosstalk between gut-liver axis and oxidative stress in metabolic dysfunction-associated steatotic liver disease. Ann Hepatol. (2025) 30(1):101777. 10.1016/j.aohep.2025.101777 39832564

[215] Chen B Wang X Pan D Wang J. Global trends and hotspots in the association between chronic kidney disease and cardiovascular diseases: a bibliometric analysis from 2010 to 2023. Cardiorenal Med. (2025) 15(1):1–20. 10.1159/000542441 39581182 PMC11844684

[216] Glick HA Orzol SM Tooley JF Remme WJ Sasayama S Pitt B. Economic evaluation of the randomized aldactone evaluation study (RALES): treatment of patients with severe heart failure. Cardiovasc Drugs Ther. (2002) 16(1):53–9. 10.1023/a:1015371616135 12085979

[217] Pitt B Williams G Remme W Martinez F Lopez-Sendon J Zannad F. The EPHESUS trial: eplerenone in patients with heart failure due to systolic dysfunction complicating acute myocardial infarction. Eplerenone post-AMI heart failure efficacy and survival study. Cardiovasc Drugs Ther. (2001) 15(1):79–87. 10.1023/a:1011119003788 11504167

[218] Rashid A Elango S Rahman P Z S. SGLT2 Inhibitors for cardioprotection. Oman Med J. (2023) 38(4):e521. 10.5001/omj.2023.128 37711978 PMC10498357

[219] Aberle J Menzen M Schmid SM Terkamp C Jaeckel E Rohwedder K. Dapagliflozin effects on haematocrit, red blood cell count and reticulocytes in insulin-treated patients with type 2 diabetes. Sci Rep. (2020) 10(1):22396. 10.1038/s41598-020-78734-z 33372185 PMC7769973

[220] Wichaiyo S Saengklub N. Alterations of sodium-hydrogen exchanger 1 function in response to SGLT2 inhibitors: what is the evidence? Heart Fail Rev. (2022) 27(6):1973–90. 10.1007/s10741-022-10220-2 35179683

